# DEEPScreen: high performance drug–target interaction prediction with convolutional neural networks using 2-D structural compound representations†
†Electronic supplementary information (ESI) available. See DOI: 10.1039/c9sc03414e


**DOI:** 10.1039/c9sc03414e

**Published:** 2020-01-08

**Authors:** Ahmet Sureyya Rifaioglu, Esra Nalbat, Volkan Atalay, Maria Jesus Martin, Rengul Cetin-Atalay, Tunca Doğan

**Affiliations:** a Department of Computer Engineering , METU , Ankara , 06800 , Turkey . Email: vatalay@metu.edu.tr ; Tel: +903122105576; b Department of Computer Engineering , İskenderun Technical University , Hatay , 31200 , Turkey; c KanSiL , Department of Health Informatics , Graduate School of Informatics , METU , Ankara , 06800 , Turkey; d European Molecular Biology Laboratory , European Bioinformatics Institute (EMBL-EBI) , Hinxton , Cambridge , CB10 1SD , UK; e Section of Pulmonary and Critical Care Medicine , The University of Chicago , Chicago , IL 60637 , USA; f Department of Computer Engineering , Hacettepe University , Ankara , 06800 , Turkey . Email: tuncadogan@hacettepe.edu.tr ; Tel: +903122977193/117; g Institute of Informatics , Hacettepe University , Ankara , 06800 , Turkey

## Abstract

The identification of physical interactions between drug candidate compounds and target biomolecules is an important process in drug discovery. Since conventional screening procedures are expensive and time consuming, computational approaches are employed to provide aid by automatically predicting novel drug–target interactions (DTIs). In this study, we propose a large-scale DTI prediction system, DEEPScreen, for early stage drug discovery, using deep convolutional neural networks. One of the main advantages of DEEPScreen is employing readily available 2-D structural representations of compounds at the input level instead of conventional descriptors that display limited performance. DEEPScreen learns complex features inherently from the 2-D representations, thus producing highly accurate predictions. The DEEPScreen system was trained for 704 target proteins (using curated bioactivity data) and finalized with rigorous hyper-parameter optimization tests. We compared the performance of DEEPScreen against the state-of-the-art on multiple benchmark datasets to indicate the effectiveness of the proposed approach and verified selected novel predictions through molecular docking analysis and literature-based validation. Finally, JAK proteins that were predicted by DEEPScreen as new targets of a well-known drug cladribine were experimentally demonstrated *in vitro* on cancer cells through STAT3 phosphorylation, which is the downstream effector protein. The DEEPScreen system can be exploited in the fields of drug discovery and repurposing for *in silico* screening of the chemogenomic space, to provide novel DTIs which can be experimentally pursued. The source code, trained "ready-to-use" prediction models, all datasets and the results of this study are available at ; https://github.com/cansyl/DEEPscreen.

## Abstract 2

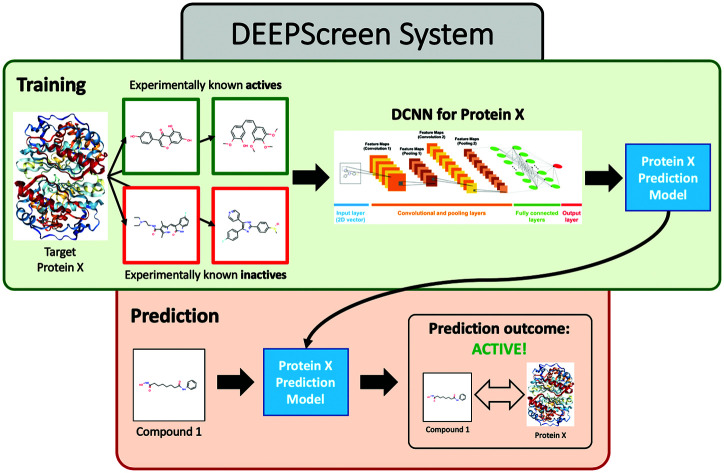
The DEEPScreen system is composed of 704 target protein specific prediction models, each independently trained using experimental bioactivity measurements against many drug candidate small molecules, and optimized according to the binding properties of the target proteins.

## 1. Introduction

One of the initial steps of drug discovery is the identification of novel drug-like compounds that interact with the predefined target proteins. *In vitro*/*in vivo* and high-throughput screening experiments are performed to detect novel compounds with the desired interactive properties. However, high costs and temporal requirements make it infeasible to scan massive target and compound spaces.^1^ Due to this reason, the rate of the identification of novel drugs has substantially been decreased.^2^ Currently, there are more than 90 million drug candidate compound records in compound and bioactivity databases such as ChEMBL^3^ and PubChem^4^ (combined), whereas the size estimation for the whole “drug-like” chemical space is around 10^60^.^5^ On the other hand, the current number of drugs (FDA approved or at the experimental stage) is around 10 000, according to DrugBank.^6^ In addition, out of the 20 000 proteins in the human proteome, less than 3000 of them are targeted by known drugs.^7^,^8^ As the statistics indicates, the current knowledge about the drug–target space is limited, and novel approaches are required to widen our knowledge. Information about the automated prediction of drug–target interactions (DTI), descriptors and feature engineering in machine learning (ML) based DTI prediction, and novel deep learning (DL) based DTI prediction approaches proposed lately in the literature are provided in the ESI, sections 1.1, 1.2 and 1.3,† respectively.

The studies published so far have indicated that DTI prediction is an open problem, where not only novel ML algorithms but also new data representation approaches are required to shed light on the un-charted parts of the DTI space^9^–^21^ and for other related tasks such as reaction^22^ and reactivity predictions^23^ and *de novo* molecular design.^24^,^25^ This effort comprises the identification of novel drug candidate compounds, as well as the repurposing of the existing drugs on the market.^26^ Additionally, in order for the DTI prediction methods to be useful in real-world drug discovery and development research, they should be made available to the research community as tools and/or services *via* open access repositories. Some examples to the available deep learning based frameworks and tools in the literature for various purposes in computational chemistry based drug discovery are given as follows: gnina, a DL framework for molecular docking (repository: ; https://github.com/gnina/gnina);^27^–^30^ Chainer Chemistry, a DL framework for chemical property prediction, based on Chainer (repository: ; https://github.com/chainer/chainer-chemistry);^31^ DeepChem, a comprehensive open-source toolchain for DL in drug discovery (repository: ; https://github.com/deepchem/deepchem);^32^ MoleculeNet, a benchmarking system for molecular machine learning, which builds on DeepChem (repository: ; http://moleculenet.ai/);^13^ and SELFIES, a sequence-based representation of semantically constrained graphs, which is applicable to represent chemical compound structures as graphs (repository: ; https://github.com/aspuru-guzik-group/selfies).^33^

In this study, we propose DEEPScreen, a deep convolutional neural network (DCNN) based a DTI prediction system that utilizes readily available 2-D structural compound representations as input features, instead of using conventional descriptors such as the molecular fingerprints.^34^ The main advantage of DEEPScreen is increasing the DTI prediction performances with the use of 2-D compound images, that is assumed to have a higher coverage in terms of compound features, compared to the conventional featurization approaches (*e.g.*, fingerprints), which have issues related to generalization over the whole DTI space.^11^,^35^ DEEPScreen system's high-performance DCNNs inherently learn these complex features from the 2-D structural drawings to produce highly accurate novel DTI predictions at a large scale. Image-based representations of drugs and drug candidate compounds reflect the natural molecular state of these small molecules (*i.e.*, atoms and bonds), which also contain the features/properties determining their physical interactions with the intended targets. Recently, image-based or similar structural representations of compounds have been incorporated as the input for predictive tasks under different contexts (*e.g.*, toxicity, solubility, and other selected biochemical and physical properties) in the general field of drug discovery and development,^35^–^38^ but have not been investigated in terms of the binary prediction of physical interactions between target proteins and drug candidate compounds, which is one of the fundamental steps in early drug discovery. In this work, we aimed to provide such an investigation, and as the output, we propose a highly optimised and practical DTI prediction system that covers a significant portion of the known bio-interaction space, with a performance that surpasses the state-of-the-art.

The proposed system, DEEPScreen, is composed of 704 predictive models; each one is independently optimized to accurately predict interacting small molecule ligands for a unique target protein. DEEPScreen has been validated and tested using various benchmarking datasets, and compared with the state-of-the-art DTI predictors using both conventional and deep ML models. Additionally, DEEPScreen target models were run on more than a million compound records in the ChEMBL database to produce large-scale novel DTIs. We also validated selected novel predictions using three different approaches: (i) from the literature, in terms of drug repurposing, (ii) with computational structural docking analysis, and (iii) *via in vitro* wet-lab experiments. Finally, we constructed DEEPScreen as a ready to use collection of predictive models and made it available through an open access repository together with all of the datasets and the results of the study at ; https://github.com/cansyl/DEEPScreen.

## 2. Results

### 2.1 Drug–target interaction prediction with DEEPScreen

**Fig. 1:**
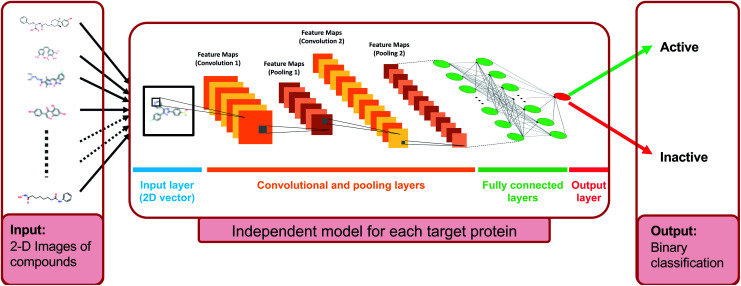
Illustration of the deep convolutional neural network structure of DEEPScreen, where the sole input is the 2-D structural images of the drugs and drug candidate compounds (generated from the SMILES representations as a data pre-processing step). Each target protein has an individual prediction model with specifically optimized hyper-parameters (please refer to the Methods section). For each query compound, the model produces a binary output either as active or inactive, considering the interaction with the corresponding target.

**Fig. 2:**
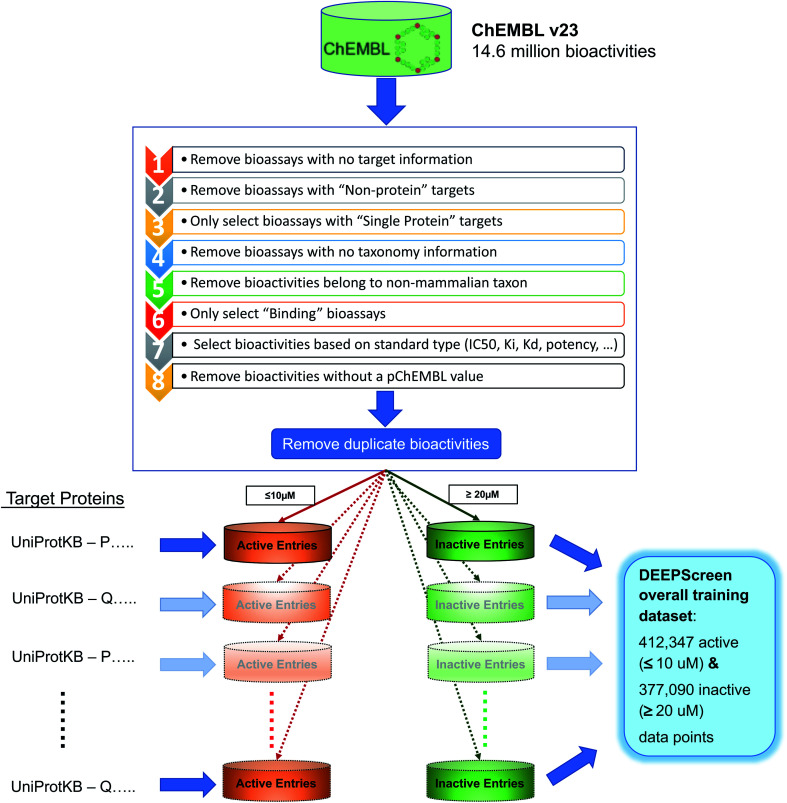
Data filtering and processing steps to create the training dataset of each target protein model. Predictive models were trained for 704 target proteins, each of which has at least 100 known active ligands in the ChEMBL database.

In this study, we approached DTI prediction as a binary classification problem. DEEPScreen is a collection of DCNNs, each of which is an individual predictor for a target protein. The system takes drugs or drug candidate compounds in the form of SMILES representations as query, generates 200-by-200 pixel 2-D structural/molecular images using SMILES, runs the predictive DCNN models on the input 2-D images, and generates binary predictions as active (*i.e.*, interacting) or inactive (*i.e.*, non-interacting) for the corresponding target protein (Fig. 1). In order to train the target specific predictive models of DEEPScreen with a reliable learning set, manually curated bio-interaction data points were obtained from the ChEMBL bioactivity database and extensively filtered (Fig. 2). The technical details regarding both the methodology and the data are given in the Methods section. Following the preparation of datasets, we extracted target protein based statistics, in terms of amino acid sequences,^7^ domains,^39^,^40^ functions, interacting compounds and disease indications.^41^,^42^ The results of this analysis can be found in ESI document section 2.1 and Fig. S1.† We also carried out several tests to examine the robustness of the DEEPScreen system against input image transformations, since this is a critical topic for CNN architectures that process 2-D images. The results of this analysis can be found in ESI document section 2.2,† together with its discussion.

### 2.2 Sources of dataset bias in model evaluation

Labelled ground-truth data are split into training/validation/test partitions in order to train, optimize and evaluate predictive models. There are two basic strategies in the field of virtual screening (or DTI prediction) in terms of dataset split. The first and the most basic one is the random-split, where the data points are separated randomly without any particular consideration. Evaluations using random-split datasets are good indicators of what would be the model performance in predicting new binders that are structurally similar (*e.g.*, containing the same scaffolds) to the compounds in the training dataset. The second widely used data split strategy in DTI prediction is the similarity-based (or non-random) split, where data points are divided according to similarities between compounds/targets/bioactivities, according to the assumed modelling approach. Here, the aim is to prevent very similar data points from ending up both in training and test sets. In ligand-based prediction approaches (such as DEEPScreen), the input samples are compounds, and as a result, datasets are split according to molecular similarities between compounds. This can be done by checking the shared scaffolds in these compounds and applying a scaffold-based split or by calculating pairwise structural similarities and clustering the compounds based on this.

There are critical points and risks in constructing training and test datasets for developing a virtual screening system and analysing its predictive performance. The first risk would be the introduction of chemical bias into the tests, where structurally similar compounds end up both in training and test datasets. This often makes the task of accurate prediction a somewhat trivial task, since structurally similar compounds usually have similar (or the same) targets. Random-split datasets usually suffer from this problem. Another risk is the negative selection bias, where negative samples (*i.e.*, inactive or non-binder compounds) in the training and/or test datasets are structurally similar to each other in a way, which is completely unrelated to their binding related properties.^43^ So, a machine learning classifier can easily exploit this feature to successfully separate them from the positives. Both of these cases would result in an overestimation of the model performance during benchmarks, especially when the tests are made to infer to performance of the models in predicting completely novel binders to the modelled target proteins. It was reported that a widely used benchmark dataset DUD-E^44^ suffers from the negative selection bias problem, even though the chemical bias issue was properly addressed during the construction of this benchmark. In DUD-E, most of the property matched decoys (*i.e.*, negatives) were found to be highly biased, as the models trained on specific targets were highly successful in identifying the negatives of completely different targets.^43^ In other words, most of the decoys shared features that make them non-binders to nearly all target proteins, and care should be taken while evaluating predictive models on this benchmark. In this study, we evaluated the performance of DEEPScreen on 5 different datasets (*e.g.*, large-scale random-split dataset, both chemical and negative selection bias free representative target dataset, ChEMBL temporal/time split dataset, MUV and DUD-E) in order to observe the behaviour of the system and its comparison with the state-of-the-art on benchmarks with differing strengths and weaknesses. The content and properties of these datasets are explained in the Methods section.

### 2.3 Analysis of the DEEPScreen dataset in terms of negative selection bias

To examine the DEEPScreen source dataset in terms of negative selection bias, we compared the average molecular similarities among the member compounds of each target specific negative training dataset; also, we make a cross comparison of average molecular similarity of the compounds in the positive training dataset a target against the compounds in the negative training dataset of the same target, to uncover if there is a statistically significant structural difference between positives and negatives. For this, we employed Morgan fingerprints (ECFP4) and the pairwise Tanimoto similarity calculation between all compound pair combinations. According to the results of this analysis of the datasets of 704 target proteins, there was no target where the inactive training dataset compounds are more similar to each other compared to the inter group similarities between the active and inactive dataset compounds of that target protein model, with statistical significance according to *t*-test (at 95% confidence interval). Actually, mean active to inactive similarity was higher than the similarity among the inactives for 211 targets, indicating that inactives do not share a global similarity that separates them from actives, which would otherwise make it easy to distinguish them, and introduce a bias into the performance analysis. These results are displayed in ESI Document Fig. S2† as target based mean pairwise compound similarity curves for intra-group (among inactives) and inter-group (actives to inactives) similarities with error bands. The most probable reason behind the observation of no significant difference was that we directly used the experimental bioassay results reported in the ChEMBL database to construct our negative datasets by setting an activity threshold (*i.e.*, ≤10 μM), instead of manually constructing decoy datasets. Thus, the compounds in our negative datasets are able to interact with the intended targets, with very low affinities. The results indicated that the negative selection bias is not an issue for the DEEPScreen source dataset.

### 2.4 Performance evaluation of DEEPScreen and comparison with other methods

#### 2.4.1 Large-scale performance evaluation and comparison with the random-split dataset

According to our basic performance tests, for 613 of the target protein models (out of 704), DEEPScreen scored an accuracy ≥0.8, with an overall average accuracy of 0.87, an *F*1-score of 0.87 and a Matthews correlation coefficient (MCC) of 0.74. Additionally, high-level target protein family based average model performances indicated that DEEPScreen performs sufficiently well on all target families (average MCC for enzymes: 0.71, GPCR: 0.80, ion channels: 0.76, nuclear receptors: 0.76, others: 0.69). All performance evaluation metrics used in this study are explained in the Methods section.

**Fig. 3:**
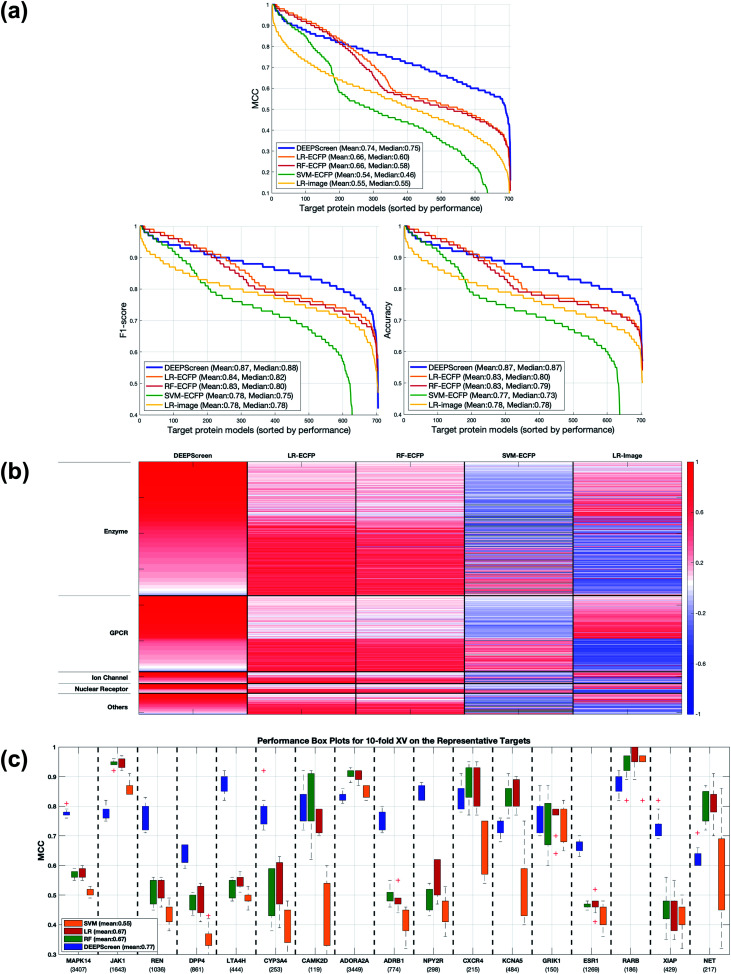
(a) Overall predictive performance comparison of DEEPScreen *vs.* state-of-the-art classifiers. Each point in the horizontal axis represents a target protein model: the vertical axis represents performance in the MCC, accuracy and *F*1-score, respectively. For each classifier, targets are ranked in a descending performance order. Average performance values (mean and median) are given inside the plots. (b) Target-based maximum predictive performance (MCC-based) heatmap for DEEPScreen and conventional classifiers (columns) (LR: logistic regression, RF: random forest, SVM: support vector machine; ECFP: fingerprint-based models, and image: 2-D structural representation-based models). For each target protein (row), classifier performances are shown in shades of red (*i.e.*, high performance) and blue (*i.e.*, low performance) colours according to *Z*-scores (*Z*-scores are calculated individually for each target). Rows are arranged in blocks according to target families. The height of a block is proportional to the number of targets in its corresponding family (enzymes: 374, GPCRs: 212, ion channels: 33, nuclear receptors: 27, and others: 58). Within each block, targets are arranged according to descending performance from top down with respect to DEEPScreen. Grey colour signifies the cases, where learning was not possible. (c) MCC performance box plots in the 10-fold cross-validation experiment, to compare DEEPScreen with the state-of-the-art DTI predictors.

Following the calculation of DEEPScreen's performance, we compared it against conventional DTI prediction approaches (classifiers: random forest – RF, support vector machines – SVM and logistic regression – LR) using the exact same random-split training/test sets under two different settings. In the first setting, conventional classifiers were trained with circular fingerprints (*i.e.*, ECFP4 (ref. 34)) of the compounds, which represents the current state-of-the-art in DTI prediction. The model parameters of the conventional classifiers were optimized on the validation dataset and the finalized performances were measured using the independent test dataset, similar to the evaluation of DEEPScreen. In the second setting, the same feature type (*i.e.*, 2-D molecular representations) is employed. These conventional classifiers normally accept 1-D (column-type) feature vectors; therefore, we flattened our 200-by-200 images to be used as the input. Thus, the performance comparison solely reflects the gain of employing DCNNs as opposed to conventional/shallow classification techniques. It is possible to argue that conventional classifiers such as LR, RF and SVM may not directly learn from the raw image features, and thus, sophisticated image pre-processing applications, such as constructing and using histograms of oriented gradients,^45^ are required to train proper image feature based predictive models. Here, our aim was to identify the most prominent factor behind the performance increase yielded by DEEPScreen (*i.e.*, is it only the use of DNNs, mostly independent from the featurization approach, or is it the use of image-based features together with the employment of DNNs to classify them), without a possible effect from a third-party data processing application. As a result, we directly used the raw image features. Fig. 3a displays the overall ranked target based predictive performance curves, in MCC, accuracy and *F*1-score, respectively. We did not include RF-Image and SVM-Image performance in Fig. 3 since RF models performed very similar to the LR models on nearly all models, and SVM models were unable to learn the hidden features in most of the cases and provided a very low performance. It is possible to observe the results of RF-Image and SVM-Image in the performance tables provided in the repository of this study. DEEPScreen performed better compared to all conventional classifiers employed in the test according to both mean and median performance measures. Especially, the performance difference was significant when the MCC was used, which is considered to be a good descriptor of DTI prediction performance. For all performance measures, among the best 200 target models for each method, LR-ECFP and RF-ECFP models have higher performance compared to DEEPScreen; however, DEEPScreen takes over after the 200^th^ model and displayed a much better performance afterwards. Overall, DEEPScreen performed 12% and 23% better in terms of mean and median performances respectively, compared to its closest competitors (*i.e.*, LR-ECFP and RF-ECFP) in terms of the MCC. According to our results, the best classifier was DEEPScreen for 356 targets (LR-ECFP for 250, RF-ECFP for 141, SVM-ECFP for 24 targets). The results indicate that DEEPScreen's performance is stable over the whole target set. On the other hand, state-of-the-art classifiers perform very well for some targets but quite bad at others, pointing out the issues related to generalization of conventional fingerprints.

Fig. 3b shows the target protein based predictive performance (in terms of the MCC) *z*-score heatmap for DEEPScreen and conventional classifiers, where each horizontal block corresponds to a target family. As displayed in Fig. 3b, DEEPScreen performed significantly better for all families (solid red blocks); LR-ECFP and RF-ECFP came second, LR-Image took the third place, and SVM-ECFP came in last place. An interesting observation here is that image-based (*i.e.*, DEEPScreen and LR-Image) and fingerprint-based classifiers display opposite trends in predictive performance for all families, indicating that the image-based approach complements the fingerprint approach. Also, LR-ECFP and LR-Image performances were mostly opposite, indicating a pronounced difference between the information obtained from fingerprints and images. Although LR-Image's overall performance was lower compared to LR-ECFP, it was still higher compared to SVM-ECFP, implying that LR-Image managed to learn at least some of the relevant hidden features. There was no significant difference between the protein families in terms of the classifier rankings; however, DEEPScreen's domination was slightly more pronounced on the families of GPCR, ion channels, and nuclear receptors.

In order to compare the performance of DEEPScreen with the conventional classifiers on a statistical basis, we carried out 10 fold cross-validation on the fundamental random-split datasets of the same 17 representative target proteins (*i.e.*, gene names: MAPK14, JAK1, REN, DPP4, LTA4H, CYP3A4, CAMK2D, ADORA2A, ADRB1, NPY2R, CXCR4, KCNA5, GRIK1, ESR1, RARB, XIAP, and NET) that were employed for the construction of a chemical and negative selection bias free scaffold-split benchmark dataset (please see Methods section for information about the selection procedure for these target proteins). We applied Bonferroni corrected *t*-tests to compare the performance distribution of each method on each target independently (10 measurements from each 10-fold cross-validation experiment constitute a distribution). The statistical tests were conducted on the MCC performance metric due to its stability under varying dataset size partitions. Fig. 3c displays the MCC performance results as box plots, for 17 targets. Each box represents a classifier's 10 MCC measures on 10 different folds of a target's training dataset, in the cross-validation. In these plots, the top and bottom borders of the box indicate the 75^th^ and 25^th^ percentiles, the whiskers show the extension of the most extreme data points that are not outliers, and plus symbols indicate outliers. The number written under the gene names of the respective targets indicates the size of the training datasets (actives). According to results, there was no observable relation between dataset sizes and a classifier's performance. According to the results of the multiple pairwise comparison test (Bonferroni corrected *t*-tests), DEEPScreen performed significantly better (compared to the best conventional classifier for each target) for 9 of the 17 representative targets (*i.e.*, genes MAPK14, REN, DPP4, LTA4H, CYP3A4, ADRB1, NPY2R, ESR1, and XIAP), which constitutes 71%, 50%, 50% and 50% of enzymes, GPCRs, nuclear receptors and ‘others’ families, respectively (*p*-value < 0.001). Whereas, the best conventional classifier managed to significantly beat DEEPScreen only for 2 representative targets (*i.e.*, genes JAK1 and RARB), which constitute 14% and 25% of enzymes and GPCRs, respectively (*p*-value < 0.001). For the rest of the representatives (6 targets), there was no statistically significant difference between DEEPScreen and the conventional classifiers. The results indicate that DEEPScreen's dominance is mostly statistically significant.

To examine the test results in relation to potential performance affecting factors, we first checked the correlation between the performances of different classifiers to observe the overlap and the complementarity between different ML algorithms and featurization approaches. Spearman rank correlation between the performance (MCC) distribution of DEEPScreen and the state-of-the-art (*i.e.*, LR, RF and SVM with fingerprint-based features) was around 0.25 (against LR-ECFP and RF-ECFP) and 0.51 (against SVM-ECFP), indicating only a slight relation and thus, a potential complementarity (as also indicated in Fig. 3B). However, the rank correlation between LR-ECFP and RF-ECFP was 0.97 indicating a high amount of overlap and possibly no complementarity. The correlation between LR-ECFP (or RF-ECFP) and SVM-ECFP was around 0.62, just slightly higher than DEEPScreen *vs.* SVM-ECFP. It was interesting to observe that DEEPScreen's performance rank was more similar to that of SVM-ECFP than LR-ECFP or RF-ECFP. To check if the difference between DEEPScreen and LR/RF is due to the employed algorithmic approach or due to the featurization approach, we checked the correlation between DEEPScreen and LR that used image features (*i.e.*, LR-Image), which resulted in a correlation value of 0.68, whereas the rank correlation between LR-ECFP and LR-Image was only 0.21. These results demonstrated that the low correlation between DEEPScreen and LR-ECFP (or RF-ECFP) was mainly due to the difference in featurization, and there is possibly a complementarity between the featurization approaches of using molecular structure fingerprints and 2-D images of compounds. Also, the observed high performance of DEEPScreen indicated that deep convolutional neural networks are successful in extracting knowledge directly from the 2-D compound images. A pairwise all-against-all Spearman rank correlation matrix is given in the ESI Table S5.†

After that, we checked if there is a relation between training dataset sizes and the performance of the models, since deep learning-based methods are often reported to work well with large training sets. For this, we calculated the Spearman rank correlation between DEEPScreen performance (MCC) and the dataset sizes of 704 target proteins, and the resulting value was –0.02, indicating no correlation. The results were similar when LR and RF were tested against the dataset sizes (–0.08 and –0.02, respectively). However, the result for SVM was 0.20, indicating a slight correlation. Finally, we checked the average dataset size of 356 target proteins, on which DEEPScreen performed better (MCC) compared to all conventional classifiers and found the mean value as 629 active compounds; we also calculated the average dataset size of the models where the state-of-the-art approaches performed better compared to DEEPScreen and found the mean value as 542 active compounds. The difference in the mean dataset sizes indicates that DEEPScreen performs generally better on larger datasets.

Next, we applied a statistical test to observe if there are significantly enriched compound scaffolds in the training datasets of target proteins, where DEEPScreen performed better compared to the state-of-the-art approaches. For this, we first extracted Murcko scaffolds^46^ of both active and inactive compounds of 704 DEEPScreen targets, using the RDkit scaffold module. Scaffold extraction resulted in a total of 114 269 unique Murcko scaffolds for 294 191 compounds. Then, we divided each scaffold's statistics into four groups: (i) the number of occurrences in the active compound datasets of targets where DEEPScreen performed better, (ii) the number of occurrences in the active compound datasets of targets where the state-of-the-art classifiers performed better, (iii) the number of occurrences in the inactive compound datasets of targets where DEEPScreen performed better, and (iv) the number of occurrences in the inactive compound datasets of targets where state-of-the-art classifiers performed better. Using these four groups, we calculated the Fisher's exact test significance (*p*-value) for the decision on the null hypothesis that there are no non-random associations between the occurrence of the corresponding scaffold in the DEEPScreen dominated target models and the state-of-the-art classifier dominated models. With a *p*-value threshold of 1 × 10^–5^, we identified 140 scaffolds, 61 of which were enriched in the DEEPScreen dominated target models. With the aim of reducing the extremely high number of unique scaffolds, we repeated the exact same procedure by using the generalized versions of the identified scaffolds. The generalization procedure (using RDkit) reduced the number of unique scaffolds to 55 813. The statistical test resulted in a total of 211 significant generalized scaffolds, 101 of which were enriched in the DEEPScreen dominated target models. Although we managed to identify several significant scaffolds, most of them were presented in the datasets of only a few targets. The most probable reason behind this was the high diversity of compounds in the DEEPScreen training datasets. SMILES representations of significant scaffolds and significant generalized scaffolds are given together with their respective *p*-values in tabular format, in the repository of DEEPScreen.

As a specific prediction example, ESI Fig. S3† displays the structural representation of Tretinoin–RXRBeta interaction, an actual approved medication, which was correctly identified by DEEPScreen during the performance tests. None of the conventional classifiers were able to predict this interaction. Tretinoin (all-trans-retinoic acid) is an anti-cancer drug used for the treatment of acute promyelocytic leukaemia (APL), among other uses. Tretinoin binds retinoic acid receptor (RAR) family proteins (agonist) to regulate multiple biological processes.^47^,^48^

#### 2.4.2 Performance evaluation and comparison of similarity-based split datasets

We compared the results of DEEPScreen with multiple state-of-the-art methods and highly novel DL-based DTI prediction approaches (please see the ESI, Section 1.3,† for more information about these methods) by employing four non-random split datasets (*i.e.*, representative targets benchmark, temporal/time split dataset, MUV and DUD-E).

##### 2.4.2.1 Comparison with the state-of-the-art using our scaffold split dataset

**Fig. 4:**
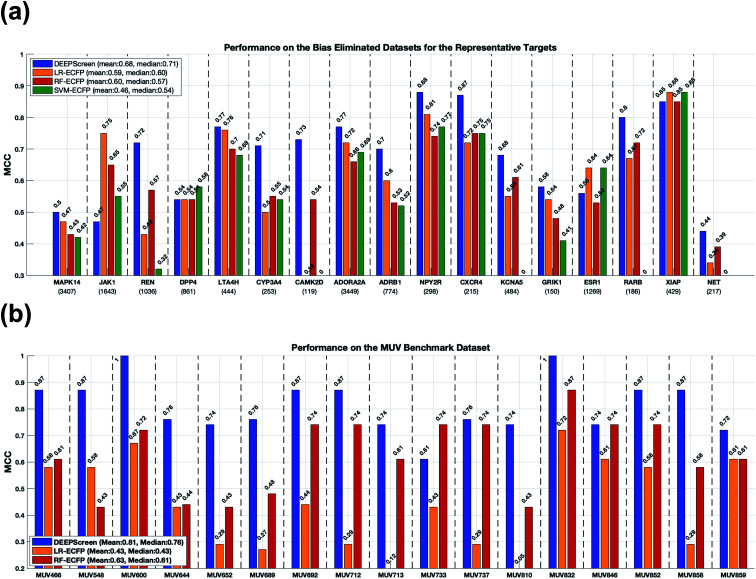
Predictive performance evaluation and comparison of DEEPScreen against the state-of-the-art DTI prediction approaches, on scaffold-split benchmarks: (a) bar plots of MCC values on representative targets dataset; (b) bar plots of MCC values on the MUV dataset.

In order to test DEEPScreen free from chemical and negative selection biases and to identify its potential to predict completely novel interacting drug candidate compounds for the intended target proteins, we carefully constructed target specific active/inactive compound datasets with a structural train-test split and collectively named it the representative target benchmark dataset (please see the Methods section for more information on this dataset). The newly constructed representative target benchmark dataset was used to train and test DEEPScreen along with the same state-of-the-art approaches used in virtual screening (*i.e.*, LR, RF and SVM with fingerprint-based features). Fig. 4a displays the performance results (MCC) on different representative targets. As observed, on average, DEEPScreen was the best performer with a median MCC of 0.71, whereas the best state-of-the-art method, LR, scored a median MCC of 0.6. RF performed similarly to LR on average and on most of the targets individually, and SVM could not manage to learn from the challenging datasets of 4 targets, where it scored MCC = 0. Out of the 17 representative targets, DEEPScreen was the best performer for 13 of them, where the combined performance of the state-of-the-art methods managed to beat DEEPScreen on 4 targets. Considering the target protein families, DEEPScreen was the best performer for 71% of the enzymes, 100% of GPCRs and ion channels, and 50% of the nuclear receptors and 'others' families. The results indicate the effectiveness of the proposed approach in terms of producing interacting compound predictions with completely different scaffolds compared to the scaffolds present in the training datasets. Chemical and negative bias eliminated representative target benchmark datasets are shared in the repository of DEEPScreen.

To benchmark DEEPScreen on an additional structural train-test split dataset and to compare it with the state-of-the-art, we employed the Maximum Unbiased Validation (MUV) dataset. Since MUV is a standard reference dataset that is frequently used to test virtual screening methods, our results are also comparable with other studies that employed the MUV benchmark. We trained DEEPScreen prediction models for 17 MUV targets using the given training split and calculated performance on the test split. We repeated the procedure using the conventional classifiers LR and RF that use fingerprint feature vectors. We left SVM out of this analysis based on its significantly inferior performance in the previous tests. The MUV performance results are shown in Fig. 4b with MCC bar plots for DEEPScreen, LR and RF. As observed from this figure, DEEPScreen had a higher performance on 15 out 17 targets, DEEPScreen and RF had the same performance on 1 target and there was a performance draw on the remaining target. Out of the 15 targets that DEEPScreen performed better on, the performance difference was highly pronounced on 14 of them. The mean MCC for DEEPScreen, LR and RF was 0.81, 0.43 and 0.63, respectively, indicating a clear performance difference on a bias free benchmark dataset.

##### 2.4.2.2 Comparison with novel DL-based DTI prediction methods using multiple benchmarks

For the DL-based DTI prediction method comparison analysis, we employed three benchmarks: temporal split, MUV and DUD-E (please refer to the Methods section for more information on these benchmark sets). We re-trained and tested DEEPScreen using the exact same experimental settings and evaluation metrics that were described in the respective articles.^11^,^18^–^20^,^49^ Two of these datasets (*i.e.*, MUV and DUD-E) are frequently employed in DTI prediction studies and the performance results of DEEPScreen on these datasets will also be comparable with future studies, where the same benchmark sets (together with the same train/test methodology) are employed. The results of this analysis reflect both the benefits of using 2-D images of compounds as the input and the constructed DCNN-based architecture. It is important to mention that in each of these benchmark tests, DEEPScreen was trained with only the training portion of the corresponding benchmark dataset (*i.e.*, MUV, DUD-E or ChEMBL temporal split set); in other words, our fundamental training dataset (Fig. 2) was not used at all. As a result, the number of training instances was significantly lower, which resulted in lower performances compared to what could have been achieved by using the regular predictive models of DEEPScreen.

Table 1 shows the results of DEEPScreen along with the performances reported in the respective articles (including both novel DL-based methods and the state-of-the-art approaches). As shown, DEEPScreen performed significantly better compared to all methods on the ChEMBL temporal split dataset. Lenselink *et al.* employed Morgan fingerprints (*i.e.*, ECFPs^34^) at the input level as the compound feature, which currently is the most widely used (state-of-the-art) ligand feature type for DTI prediction. On their temporal split test dataset, DEEPScreen performed 36% better compared to the best model in the study by Lenselink *et al.* (*i.e.*, multi-task DNN PCM – proteochemometics, also a deep learning based classifier), indicating the effectiveness of employing 2-D image-based representations as input features.

**Table 1:** The average predictive performance comparison between DEEPScreen and various novel DL-based and conventional DTI predictors

Dataset	Reference	Method/architecture	Performance (metric)
ChEMBL temporal-split dataset		DEEPScreen: DCNN with 2-D images	**0.45 (MCC)**

	Lenselink *et al.*^18^	Feed-forward DNN PCM (best model)	0.33 (MCC)
		Feed-forward DNN	0.30 (MCC)
		SVM	0.29 (MCC)
		LR	0.26 (MCC)
		RF	0.26 (MCC)
		Naïve Bayes	0.10 (MCC)

Maximum unbiased validation (MUV) dataset		DEEPScreen: DCNN with 2-D images	**0.88 (AUROC)**

	Kearnes *et al.*^11^	Graph convolution NNs (W_2_N_2_)	0.85 (AUROC)

	Ramsundar *et al.*^49^	Pyramidal multitask neural net (PMTNN)	0.84 (AUROC)
		Multitask neural net (MTNN)	0.80 (AUROC)
		Single-task neural net (STNN)	0.73 (AUROC)
		RF	0.77 (AUROC)
		LR	0.75 (AUROC)

DEEPScreen was the best performer on the MUV dataset (Table 1), by a small margin, compared to the graph convolutional neural network (GCNN) architecture proposed by Kearnes *et al.*^11^ It is interesting to compare DEEPScreen with GCNN models since both methods directly utilize the ligand atoms and their bonding information at the input level, with different technical featurization strategies. Nevertheless, the classification performance of both methods on the MUV dataset was extremely high and more challenging benchmark datasets are required to analyse their differences comprehensively. The performance difference between DEEPScreen (or GCNN) and most of the DL-based methods with conventional features such as the molecular fingerprints (as employed in Ramsundar *et al.*^49^) indicate the improvement yielded by novel featurization approaches. It is also important to note that the performance results given for LR and RF on the MUV results section of Table 1 were calculated by Ramsundar *et al.*; however, LR and RF MUV benchmark results that we provided in Fig. 4b were calculated by us.

We also tested DEEPScreen on the DUD-E dataset and obtained a mean performance of 0.85 area under receiver operating characteristic curve (AUROC). DTI prediction methods utilizing 3-D structural information such as AtomNet^19^ and those reported by Gonczarek *et al.*^20^ and Ragoza *et al.*^28^ also employed this dataset and achieved similar predictive performances. However, their results are not directly comparable with DEEPScreen since these methods utilize both target and ligand information at the input level and reserved some of the targets (along with their ligand information) for the test split during the performance analysis. Also, structure-based methods are usually benchmarked by their success in ranking several docking poses and/or success in minimizing the atomic distances from native binding poses, instead of providing binary predictions as active/inactive. It is important to note that the methods employing 3-D structural features of the target proteins may provide better representations to model DTIs at the molecular level; however, they are highly computationally intensive. Also, 3-D structural information (especially the target–ligand complexes) is only available for a small portion of the DTI space; as a result, their coverage is comparably low and they generally are not suitable for large-scale DTI prediction. It is also important to note that the DUD-E benchmark dataset is reported to suffer from negative selection bias problem,^43^ and thus, the results based on this dataset may not be conclusive.

Next, we demonstrated the predictive potential of DEEPScreen by two case studies through *in vitro* experimentation and molecular docking case studies.

### 2.5 *In vitro* validation of JAK proteins as DEEPScreen predicted cladribine targets

Cladribine (2-chlorodeoxyadenosine (2-CDA)) is a well-known purine nucleoside analog which is approved as an anti-neoplastic agent in some of forms of lymphoma, leukemia and immunosuppressive drug in multiple sclerosis.^50^,^51^ In this analysis, we predicted a set of protein targets for cladribine with the DEEPScreen system, as a case study. JAK1, JAK2 and JAK3 were on the prediction list (Table S4†), none of which were previously reported to be the target of cladribine, to the best of our knowledge albeit there are studies indicating the involvement STAT protein phosphorylation with cladribine treatment in multiple myeloma cells.^52^,^53^ Since JAK/STAT signaling was involved in both lymphoblastic diseases and immune response and since it has been previously reported that it might be involved in cladribine action, we pursued to validate cladribine and JAK/STAT DEEPScreen prediction *in vitro*.

**Fig. 5:**
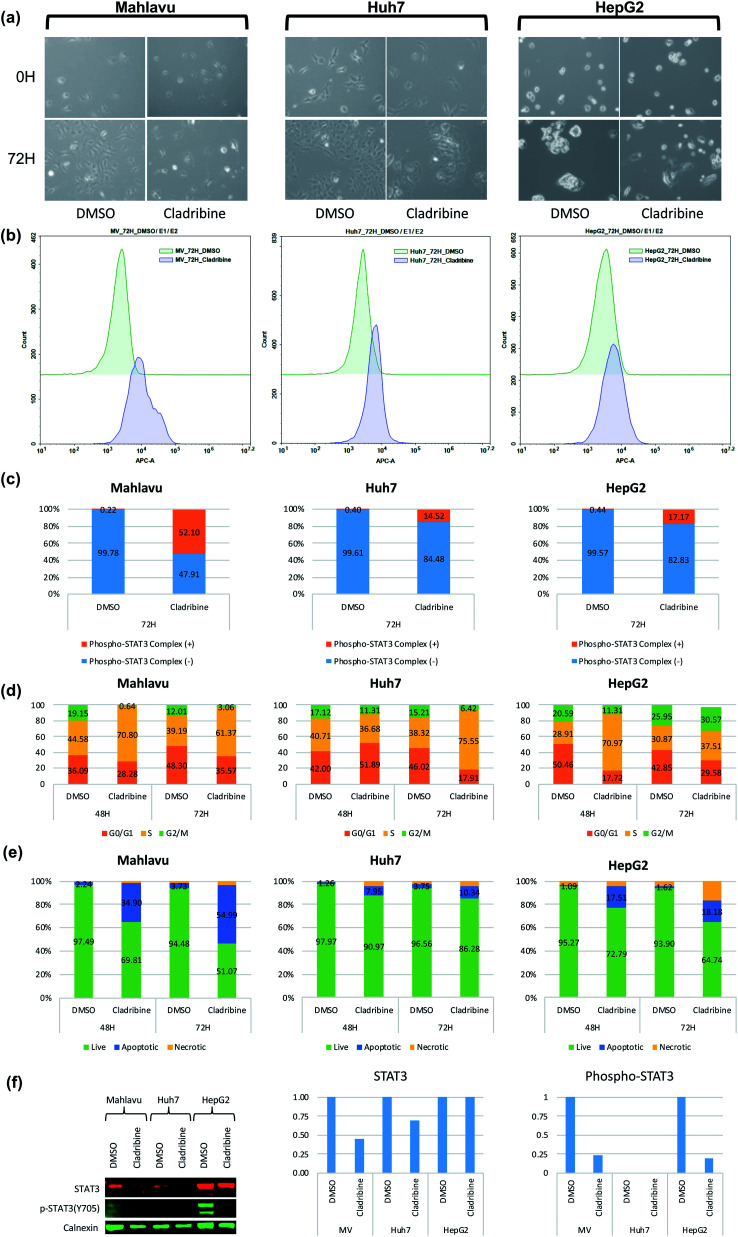
JAK downstream effector alteration in the presence of cladribine. (a) Live cell images for cladribine treated cells before (0H) and after 72 hours of treatment (72H). (b) Flow cytometry histogram of the phosphorylated STAT3 protein complex in Mahlavu, Huh7 and HepG2 cells. (c) STAT3 protein complex levels in Mahlavu, Huh7 and HepG2 cells detected and assessed with Phospho-Tyr705 antibodies. (d) Cell cycle analysis: (e) apoptotic cells characterized by annexin V assay. (f) Changes in protein expression levels of STAT3 related to cladribine treatment. Bar graphs represent normalized STAT3 and phospho-STAT3 compared to calnexin. DMSO was used as the vehicle control.

The Janus kinase/signaling transducers and activators of the transcription (JAK/STAT) pathway, activated by cytokines and growth factors, play important roles in the immune system, cell survival, cell proliferation and cell death, and tumor development.^54^ The signal transducer and activator of transcription 3 (STAT3) is one of the downstream effectors of JAK proteins. Upon JAK stimulation, STAT3 is phosphorylated and acts as the transcription activator. Initially cytotoxic activities of cladribine were assessed on hepatocellular carcinoma cell lines, Huh7, HepG2, and Mahlavu, which were reported to have adequate JAK signaling.^55^ IC_50_ values of cladribine on HCC cells (3 μM, 0.1 μM, and 0.4 μM for Huh7, HepG2, and Mahlavu cells, respectively) demonstrated that cladribine displays cytotoxic bioactivities on these cells (Table S3†). We then tested the effect of cladribine on the phosphorylation of the downstream effector protein STAT3, in order to validate our interaction prediction. Our data with cladribine treated HCC cells clearly demonstrated an alteration in phosphorylation of the STAT3 complex associated signal in flow cytometry (14.5%, 52%, and 17% in Huh7, Mahlavu and HepG2, respectively), when compared to DMSO controls (Fig. 5c). The changes of protein levels of STAT3 were also controlled with protein electrophoresis (Fig. 5f). It is a well-known fact for immune cells that the activation of STAT3 induces the expression of proapoptotic genes such as caspase and induces apoptosis.^56^ Also, there are studies stating that activation of JAK/STAT3 signaling through cytokines induce programmed cell death.^57^ We also demonstrated that cladribine treatment leads to apoptotic cell death with G1/S phase cell cycle arrest (Fig. 5d and e) and finally, a direct STAT3 phosphorylation at tyrosine 705 upon cladribine treatment. DEEPScreen predictions for cladribine identified JAK proteins as candidate targets of this well-known drug, and our experimental data validated that cladribine acts on JAK/STAT3 signaling and induces apoptosis in HCC cells.

### 2.6 DEEPScreen predicts new small molecules potentially acting on renin protein

**Fig. 6:**
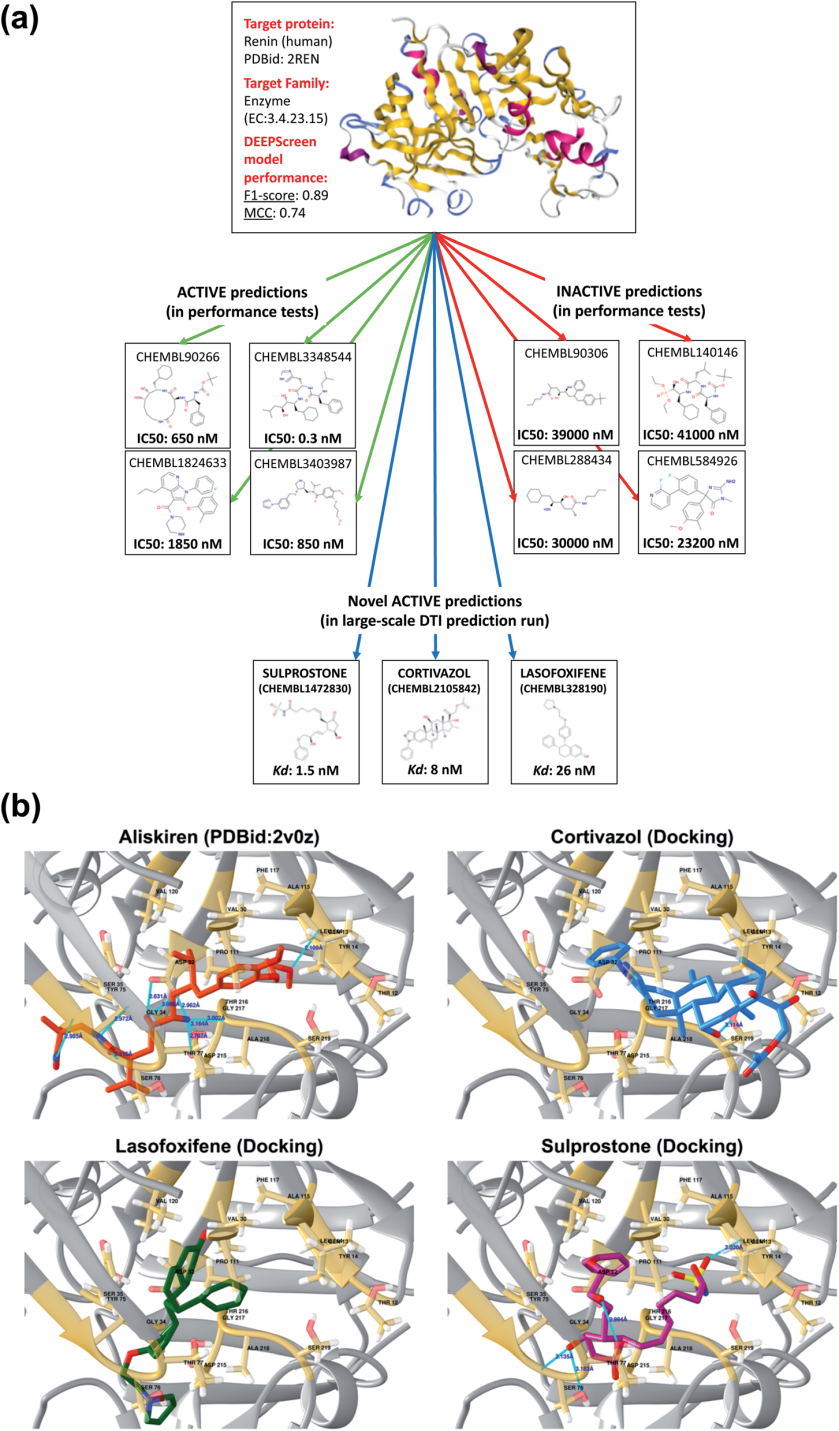
A case study for the evaluation of DEEPScreen predictions. (a) 3-D structure of the human renin protein (obtained from PDB id: 2REN), together with the 2-D representations of selected active (connected by green arrows) and inactive (connected by red arrows) ligand predictions in the predictive performance tests (the true experimental screening assay activities – IC_50_ – are shown under the corresponding images). Also, 2-D images of selected truly novel predicted inhibitors of renin (*i.e.*, cortivazol, lasofoxifene and sulprostone) are displayed (connected by blue arrows) together with the estimated docking *K*_d_ values. (b) Renin–aliskiren crystal structure (PDB id: ; 2V0Z, aliskiren is displayed in red color) and the best poses in the automated molecular docking of DEEPScreen predicted inhibitors of renin: cortivazol (blue), lasofoxifene (green) and sulprostone (violet), to the structurally known binding site of renin (gold color), displaying hydrogen bonds with light blue lines. The docking process produced sufficiently low binding free energies for the novel inhibitors, around the levels of the structurally characterized ligands of renin, aliskiren and remikiren, indicating high potency.

To further indicate that DEEPScreen is able to identify new potential inhibitors for the modelled target proteins, we conducted a molecular docking-based case study on human renin protein. Renin is an enzyme that generates angiotensin I from angiotensinogen in the plasma, as a part of the renin–angiotensin–aldosterone hormonal system (RAAS).^58^ Renin is targeted using small molecule inhibitors, with the aim of regulating arterial blood pressure (*e.g.*, Aliskiren, an approved drug licensed to treat hypertension).^59^,^60^ Studies suggest the requirement of novel renin inhibitors due to reported cases of hyperkalaemia and acute kidney injury in both mono and combination therapies of the approved/investigational renin and other RAAS system members' inhibitors.^61^ In order to propose new potential renin inhibitors, we run the DEEPScreen human renin protein model on nearly 10 000 approved/investigational small molecule drugs recorded in the DrugBank database, 795 of which have been predicted as interacting. For docking, we randomly selected drugs from this prediction set as cortivazol (glucocorticoid, investigational drug), misoprostol (prostaglandin, approved drug), lasofoxifene (estrogen receptor modulator, approved drug) and sulprostone (prostaglandin, investigational drug). As far as we are aware, the predicted drug molecules have never been screened against renin *via in silico*, *in vitro* or *in vivo* assays. We also docked two molecules with known crystal complex structures with renin, which were aliskiren and remikiren, as reference for the binding energy comparison with the predicted molecule dockings. The binding free energies (Δ*G*) of aliskiren and remikiren were estimated to be –13.9 and –10.5 kcal mol^–1^ (*K*_d_ ≈ 0.06 and 19 nM) at their best pose, respectively. The Δ*G* values of cortivazol, lasofoxifene, misoprostol and sulprostone were estimated to be –11.4, –10.5, –9.1 and –12.1 kcal mol^–1^ (*K*_d_ ≈ 4.1, 18.9, 202 and 1.3 nM), respectively. In Fig. 6, active/inactive test dataset predictions and selected completely novel inhibitor predictions (*i.e.*, cortivazol, lasofoxifene and sulprostone) for human renin protein are shown along with the best poses in their docking with the renin binding site.

In order to further validate the selected new prediction results, we randomly selected 4 drug molecules from the set of inactive (*i.e.*, non-interacting) predictions of the renin target protein model and carried out molecular docking analysis using the exact same procedure applied for the active predictions of renin. The molecules randomly selected for docking were acetylsalicylic acid – aspirin (cyclooxygenase inhibitor, approved drug), calcifediol (vitamin D receptor agonist, approved drug), difluprednate (glucocorticoid receptor agonist, approved drug) and mivacurium (muscle-type nicotinic acetylcholine receptor antagonist, approved drug). The docking binding free energies (Δ*G*) were found to be –5.8, –9.5, –8.9 and –6.7 kcal mol^–1^ for acetylsalicylic acid, calcifediol, difluprednate and mivacurium, respectively. As indicated by the high binding free energy measurements for acetylsalicylic acid, difluprednate and mivacurium, the negative predictions are validated in three out of four cases. For calcifediol, it was not possible to reach a clear conclusion since the resulting binding free energy was close to a generally accepted rough threshold to assume a potential activity (*i.e.*, –10 kcal mol^–1^). The results of the docking analysis indicate that DEEPScreen has the potential to predict novel inhibitors for renin with predicted potencies around the levels of its approved/investigational drug ligands (in 3 out of 4 selected cases). However, extensive further investigation is required to verify these results and to indicate that these predicted small molecules can actually bind renin, since docking analysis alone cannot reliably represent binding.

### 2.7 Large-scale production of the novel DTI predictions with DEEPScreen

The DEEPScreen system was applied to more than a million small molecule compound records in the ChEMBL database (v24) for the large-scale production of novel DTI predictions. As a result of this run, a total of 21 481 909 DTIs were produced (*i.e.*, active bio-interaction predictions) between 1 339 697 compounds and 532 targets. Out of these, 21 151 185 DTIs between 1 308 543 compounds and 532 targets were completely new data points, meaning that they are not recorded in ChEMBL v24 (the prediction results are available in the repository of DEEPScreen). Apart from this, newly designed compounds that are yet to be recorded in the ChEMBL database can also be queried against the modelled targets using the stand alone DEEPScreen models available in the same repository.

We carried out a statistical analysis in order to gain an insight into the properties of the compounds predicted for the members of the high level protein families in the large-scale DTI prediction set. For this, an ontology based enrichment test was conducted (*i.e.*, drug/compound set enrichment) to observe the common properties of the predicted compounds. In the enrichment analysis, over-represented annotations (in terms of ontology terms) are identified for a query set and ranked in terms of statistical significance.^62^ The enrichment tests was done for ChEBI structure and role definitions,^63^ chemical structure classifications and ATC (Anatomical Therapeutic Chemical Classification System) codes,^64^ together with experimentally known target protein and protein family information of the predicted compounds (source: ChEMBL, PubChem and DrugBank), functions of these experimentally known target protein and families (Gene Ontology^65^), and disease indications of these experimentally known target protein and families (MESH terms^66^ and Disease Ontology^67^). Multiple online tools have been used for this analysis: CSgator,^62^ BiNChE^68^ and DrugPattern.^69^

Since the compounds in the query sets have to be annotated with the abovementioned ontology based property defining terms, we were able to conduct this analysis on a subset of the compounds in the DTI prediction set (*i.e.*, nearly 30 000 ChEMBL compounds for ChEBI ontology and 10 000 small molecule drugs from DrugBank v5.1.1 for the rest of the ontology types, with a significant amount of overlap between these two). The overall prediction set used in the enrichment analysis was composed of 377 250 predictions between these 31 928 annotated compounds and 531 target proteins. It was not possible to carry out an individual enrichment analysis for the predicted ligand set of each target protein due to a high number of targets (*i.e.*, 704). Instead, we analyzed the ligand set predicted for each target protein family (*i.e.*, enzymes, GPCRs, nuclear receptors, ion channels and others) together with an individual protein case study considering the renin protein. For each protein family, the most frequently predicted 100 compounds, each of which has been predicted as active for more than 10% of the individual members of the respective target family, are selected and given as input to the enrichment analysis (*i.e.*, a compound should be annotated to at least 38 enzymes in order to be included in the enrichment analysis set of the enzymes, since there are 374 enzymes in total). The reason behind not using all predicted compounds was that there were a high number of compounds predicted for only 1 or 2 members of a target family, which add noise to the analysis when included. ChEMBL ids of the compounds predicted for each target family are given in the repository of the study together with their prediction frequencies.

The results of the enrichment analysis are shown in Table 2, where rows correspond to target protein families and columns correspond to different ontology types. For each protein family – ontology type combination, selected examples from the most enriched terms are given considering *p*-values, which are calculated as described in the respective papers of CSgator, BiNChE and DrugPattern tools. In the cases of numerous enriched terms existing, representative terms were selected from a group of closely related enriched ontological terms, as shown in Table 2. The first observation from Table 2 is the high correspondence between the predicted and experimentally identified known target families, which indicates that there is a small amount of cross protein family target transfer in DEEPScreen predictions. Compared to the rest of the target families, the enriched experimentally identified known targets of the predicted drug set of the “others” family have high variance, since the proteins in this family are coming from multiple protein families of small sizes. The structure classes are mostly distinct for the compound sets predicted for different target families, which can be observed from the columns entitled “ChEBI structure classification” and “Chemical structure classification”. Being a member of the enzyme family (*i.e.*, an aspartic protease), renin's predicted interacting compounds' enriched properties are similar to that of enzymes'. One interesting observation here is that the enriched experimentally identified known target families for the predicted drug set of renin include sub-families of kinases, which indicates a transfer of kinase inhibitors to proteases that can be utilized for drug repurposing. Disease indication columns show the enriched disease records that are associated with the experimentally identified known targets of the predicted drug sets. Considering renin's drug set predictions' enriched diseases, two of them are “cardiovascular diseases” and “vascular diseases”, two generic disease groups, where one of the members is hypertension. This finding indirectly validates the predictions since renin is targeted to treat hypertension.^70^ The other enriched disease groups indicate that some of the drugs currently used as medication for cancers, digestive system diseases and urinary system diseases may have a potential to be repurposed to target renin.

**Table 2:** Target protein family based drug/compound enrichment analysis results of the large-scale DTI predictions

Drug sets predicted to	Enriched terms of the predicted drug sets						
	ChEBI structure classification (tool: BiNChE)	Chemical structure classification (tool: DrugPattern)	ATC codes (tool: DrugPattern)	Experimentally identified known targets and families (tool: CSgator)	Functions of the experimentally identified known targets (GO) (tool: CSgator)	Disease indications (MeSH) (tool: CSgator)	Disease indications (disease ontology) (tool: CSgator)
Enzymes	CHEBI:133004 – bisbenzylisoquinoline alkaloid	Indoles and derivatives	Antineoplastic agents	Kinase	Phosphorylation (GO:0016310)	Neoplasms (C04 (D009369))	Disease of cellular proliferation (DOID:14566)
	CHEBI:50047 – organic amino compound	Macrolides and Analogues	Antivirals for systemic use	Enzyme	Cellular protein modification process (GO:0006464)	Cardiovascular diseases (C14 (D002318))	Organ system cancer (DOID:0050686)
	CHEBI:24698 – hydroxyflavone		Drugs for obstructive airway diseases	Camk (CAMK protein kinase group)	Receptor signaling protein activity (GO:0005057)	Urologic diseases (C12.777 (D014570))	Kidney disease (DOID:557)
	CHEBI:24921 – isoquinoline alkaloid			Ste (STE protein kinase group)	Transferase activity (GO:0016740)	Kidney diseases (C12.777.419 (D007674))	Urinary system disease (DOID:18)
					Adenyl nucleotide binding (GO:0030554)		

GPCRs	CHEBI:33853 – phenols	Morphinans	Psycholeptics	Small molecule receptor (family A GPCR)	Cell–cell signaling (GO:0007267)	Nervous system diseases (C10 (D009422))	Disease of mental health (DOID:150)
	CHEBI:72720 – flavonoid oligomer	Benzofurans	Drugs for obstructive airway diseases	Transporter	Circulatory system process (GO:0003013)	Cardiovascular diseases (C14 (D002318))	Central nervous system disease (DOID:331)
	CHEBI:33822 – organic hydroxy compound	Stilbenes	Bile and liver therapy	7TM1 (family A G protein-coupled receptor)	Plasma membrane region (GO:0098590)	Neurologic manifestations (C23.888.592 (D009461))	Genetic disease (DOID:630)
	CHEBI:33635 – polycyclic compound		Antihistamines for systemic use	Electrochemical transporter	Transmembrane transporter activity (GO:0022857)	Pituitary diseases (C19.700 (D010900))	Disease of metabolism (DOID:0014667)

Nuclear receptors	CHEBI:51958 – organic polycyclic compound	Steroids and steroid derivatives	Antineoplastic agents	Transcription factor	Intracellular receptor signaling pathway (GO:0030522)	Neoplasms (C04 (D009369))	Disease of cellular proliferation (DOID:14566)
	CHEBI:33635 – polycyclic compound	Morphinans	Corticosteroids for systemic use	Nuclear receptor	Nucleic acid binding transcription factor activity (GO:0001071)	Immune system diseases (C20 (D007154))	Cancer (DOID:162)
	CHEBI:36615 – triterpenoid	Prenol lipids	Anti-acne preparations	Cytochrome P450	Programmed cell death (GO:0012501)	Hemic and lymphatic diseases (C15 (D006425))	Immune system disease (DOID:2914)
	CHEBI:25872 – pentacyclic triterpenoid	Stilbenes	Analgesics	AUXTRANS (Auxiliary transport protein)	Positive regulation of apoptotic process (GO:0043065)	Skin and connective tissue diseases (C17 (D017437))	Hypersensitivity reaction disease (DOID:0060056)
			Sex hormones and modulators of the genital system				

Ion channels	CHEBI:47916 – flavonoid	Morphinans	Antihistamines for systemic use	Ste (STE protein kinase group)	Phosphorylation (GO:0016310)	Neoplasms (C04 (D009369))	Disease of cellular proliferation (DOID:14566)
	CHEBI:26004 – phenylpropanoid	Benzopyrans	Beta blocking agents	Camk (CAMK protein kinase group)	Receptor signaling protein activity (GO:0005057)	Skin and connective tissue diseases (C17 (D017437))	Organ system cancer (DOID:0050686)
	CHEBI:26195 – polyphenol	Benzene and substituted derivatives	Drugs for obstructive airway diseases	Ion channel	Transmembrane transport (GO:0055085)	Neurologic manifestations (C10.597 (D009461))	Gastrointestinal system disease (DOID:77)
	CHEBI:38443 – 1-benzopyran		Analgesics	Transporter	Cell death (GO:0008219)	Respiratory tract diseases (C08 (D012140))	Cardiovascular system disease (DOID:1287)

Others	CHEBI:33822 – organic hydroxy compound	Macrolactams	Antimycobacterials	Cytosolic other	Proteasome complex (GO:0000502)	Cardiovascular diseases (C14 (D002318))	Disease of anatomical entity (DOID:7)
	CHEBI:26004 – phenylpropanoid	Diazines	Agents acting on the renin–angiotensin system	ATP binding cassette	Ribosome (GO:0005840)	Nervous system diseases (C10 (D009422))	Cardiovascular system disease (DOID:1287)
	CHEBI:47916 – flavonoid	Carboxylic acids and derivatives	Lipid modifying agents	Cytochrome P450	Antigen processing and presentation (GO:0019882)	Vascular diseases (C14.907 (D014652))	Disease of cellular proliferation (DOID:14566)
	CHEBI:25036 – lignan		Antivirals for systemic use	Transporter	Enzyme inhibitor activity (GO:0004857)	Pathological conditions, signs and symptoms (C23 (D013568))	Primary bacterial infectious disease (DOID:0050338)
				Phosphodiesterase			

Renin (case study)	CHEBI:35352 – organonitrogen compound	Morphinans	Antineoplastic agents	Kinase	Phosphorylation (GO:0016310)	Neoplasms (C04 (D009369))	Disease of cellular proliferation (DOID:14566)
	CHEBI:26188 – polyketide	Macrolides and analogues	Pituitary and hypothalamic hormones and analogues	Tk (TK protein kinase group)	Receptor signaling protein activity (GO:0005057)	Cardiovascular diseases (C14 (D002318))	Organ system cancer (DOID:0050686)
	CHEBI:46845 – *N*-alkylpiperazine	Lactams	Endocrine therapy	Cmgc (CMGC protein kinase group)	Adenyl nucleotide binding (GO:0030554)	Vascular diseases (C14.907 (D014652))	Vascular disease (DOID:178)
	CHEBI:37578 – halide	Benzodiazepines		Agc (AGC protein kinase group)	Guanyl-nucleotide exchange factor activity (GO:0005085)	Digestive system diseases (C06 (D004066))	Urinary system disease (DOID:18)

### 2.8 Literature based validation of novel DTI predictions towards drug repurposing

With the aim of evaluating novel predictions, we conducted a literature-based search to find evidence on selected predicted DTIs. In this analysis, we focused on the recently discovered human target protein interactions of already approved (or investigational) drugs to show that DEEPScreen can be utilized towards drug repurposing. Table 3 displays the literature-validated DTI predictions together with the source publication for each interaction. In Table 3, a few inactive (*i.e.*, non-interacting) predictions are given along with many active ones. The reason behind the imbalance between active and inactive cases is that the inactive/negative results are usually not reported in the literature. We also included (at the end of Table 3) 4 correct prediction cases, where completely new compounds are tested against selected targets. All of the bio-interactions shown in Table 3 are released (either in ChEMBL v24 or in the literature) at least 6 months after the training of DEEPScreen. As a result, they were completely unknown and novel according to the DEEPScreen models. Nevertheless, they were correctly predicted.

**Table 3:** Literature verified selected DTI predictions of DEEPScreen

Ligand (drug/compound)	Target protein	DEEPScreen prediction	Experimental bioactivity	Reference
Fedratinib	Bromodomain-containing protein 4 – BRD4 (O60885)	Active	IC_50_: 290 nM	71

Hydralazine	Myeloperoxidase – MPO (P05164)	Active	IC_50_: 900 nM	72

Varlitinib	Receptor protein-tyrosine kinase erbB-2 (P04626)	Active	IC_50_: 2 nM	73

Armodafinil	D(2) dopamine receptor – DRD2 (P14416)	Active	IC_50_: 2.1 nM	74
	D(3) dopamine receptor – DRD3 (P35462)	Inactive	IC_50_: 39 000 nM	

Copanlisib	Phosphatidylinositol 4,5-bisphosphate 3-kinase catalytic subunit beta isoform – PIK3CB (P42338)	Active	IC_50_: 3.7 nM	75

Dacomitinib	Tyrosine-protein kinase Lck (P06239)	Active	IC_50_: 94 nM	76

Encorafenib	Serine/threonine-protein kinase B-raf (P15056)	Active	IC_50_: 0.3 nM	77

Prednisolone	Progesterone receptor – PGR (P06401)	Active	IC_50_: 2080 nM	78

Apratastat	Stromelysin-1/matrix metalloproteinase 3 (P08254)	Active	IC_50_: 10 nM	79 and 80
	Collagenase/matrix metalloproteinase 9 (P14780)	Active	IC_50_: 82 nM	

CEP-37440 (CHEMBL3951811)	ALK tyrosine kinase receptor (Q9UM73)	Active	IC_50_: 3.1 nM	81
	Insulin receptor (P06213)	Active	IC_50_: 65 nM	
	Focal adhesion kinase 1 – PTK2 (Q05397)	Active	IC_50_: 2 nM	

Ketotifen	Histamine H4 receptor – HRH4 (Q9H3N8)	Inactive	IC_50_: 21 000 nM	82 and 83

INH14 (*N*-(4-ethyl phenyl)-*N*′-phenyl urea)	Inhibitor of nuclear factor kappa-B kinase subunit beta – IKBKB (O14920)	Active	IC_50_: 3590 nM	84

2-Allyl-7-chloro-1*H*-indole-3-carbonitrile	Dual specificity protein kinase CLK4 (Q9HAZ1)	Active	IC_50_: 533 nM	85

7-Bromo-2-phenyl-1*H*-indole-3-carbonitrile	Dual-specificity tyrosine-phosphorylation regulated kinase 1A – DYRK1A (Q13627)	Active	IC_50_: 25 nM	

7-Iodo-2-(3-methoxyphenyl)-1*H*-indole-3-carbonitrile	Dual-specificity tyrosine-phosphorylation regulated kinase 2 – DYRK2 (Q92630)	Inactive	IC_50_ > 10 000 nM	

## 3. Discussion

In this study, we proposed DEEPScreen, a novel deep learning based drug/compound-target prediction system. The major contributions of DEEPScreen to the literature can be listed as follows:

(i) the idea of using compound images for predicting the interactions with target proteins and employing established convolutional neural network architectures that showed high performance in image recognition/analysis tasks;

(ii) constructing (and open access sharing) a reliable experimental DTI dataset to be used as training/test sets, both in this study and in other future studies. The existing reference DTI datasets are usually small-scale; thus, there is a requirement for high quality large-scale datasets especially for deep learning based model training;

(iii) generating highly optimized, high performance predictive models for 704 different target proteins, each of which was independently trained and optimized with rigorous tests. This approach gave way to a significant performance improvement over the state-of-the-art;

(iv) conducting a high number of experiments and data analysis processes in terms of benchmarks/performance tests and comparisons with the state of the art to understand the model/system behavior under different conditions.

(v) publishing the method as an open access tool. DEEPScreen is practical to use since it is composed of independent modules (*i.e.*, each target protein model), where only the model of the target of interest should be downloaded and run to produce predictions;

(vi) executing a systematic large-scale DTI prediction run between 704 targets and 1.3 million drug candidate compounds recorded in the ChEMBL database. Selected examples from the novel predictions have been tested and validated by molecular docking analysis and *in vitro* experiments on cancer cells for potential future drug discovery and repurposing applications.

The main reason why DEEPScreen works better compared to the state-of-the-art DTI prediction approach is that molecular descriptors such as fingerprints make assumptions regarding what parts in a molecule are important for target binding and generate feature vectors for storing the information of the presence or absence of these groups (*i.e.*, feature engineering); thus, the information that is deemed unimportant for binding is eliminated. As such, the ML predictor is provided only with a limited piece of information to work with. Besides, it is not possible to generalize these assumptions to the whole DTI space, which is indicated by the limited predictive performance obtained with the conventional approach. By employing 2-D structures generated from SMILES, the system does not make any prior assumptions and just provides a vector displaying the entire molecule with a representation similar to its state in nature, to let the DCNN identify the parts necessary for the interaction with the corresponding target protein. Provided with a sufficient number and structural variety of active data points, DEEPScreen was able to learn the relevant interactive properties and provided accurate DTI predictions. Based on the performance results obtained in this study, it is possible to state that the performance improvement of DEEPScreen comes from both using image features and a deep learning approach that is suitable to extract information from images. It is possible that adding the 3-D representations of molecules (*i.e.*, conformational information) to the system would provide a more accurate modelling; however, DCNNs that employ 3-D convolutions are computationally highly intensive, which prevents practical applications at a large scale.

In DEEPScreen, we modelled the interactive properties of each target protein independently in a separate DCNN. This allowed the learning of target specific binding properties during the training process (*i.e.*, the optimization of hyper-parameters and the regular model parameters). In most of the ML method development studies, hyper-parameters are arbitrarily pre-selected without further optimization (especially when there are a high number of models as in the case of DEEPScreen), due to extremely high computational burden. However, hyper-parameters are an important part of the model architecture and significantly contribute to the predictive performance. In this study, we evaluated hundreds to thousands of models for each target, resulting in more than 100 000 model training and evaluation jobs in total (considering the hyper-parameter value options in Table S1† and their combinations with each other). As a result, a strong computing cluster and extensive levels of parallelization were required to practically run the computational jobs. Whereas, the main advantage of this approach is the elevated predictive performance, which was indicated by the results of the performance tests.

An important concern in ML method development is the problem of overfitting. We employed the neuron drop-out technique, a widely accepted approach for DCNN training, in order to prevent this issue. The results of the independent tests and benchmarking experiments confirmed that overfitting was not a problem for DEEPScreen. Further discussion about the DEEPScreen system has been provided in the ESI, Section 2.†

One direction in which DEEPScreen can be improved would be the incorporation of target proteins with only a few known small molecule interactions and the ones without any (*i.e.*, target discovery). DEEPScreen only takes the features of compounds at the input level and treats the target proteins as labels, which allowed ligand predictions for only 704 highly-studied proteins (*i.e.*, the major limitation of DEEPScreen). Within a multi-task modelling approach, targets with only a few known interactions can be incorporated together with the well-studied targets. In this scheme, data augmentation techniques can be incorporated such as generative adversarial networks to balance the training datasets. To be able to provide predictions for proteins without known interactions, target descriptors may be incorporated at the input level along with compound features, within a chemogenomic modelling approach. Image or graph based structural representations of proteins can be used for this purpose.

## 4. Methods

### 4.1 Generation of the fundamental training dataset

The ChEMBL database (v23) was employed to create the training dataset of DEEPScreen. There are 14 675 320 data points (*i.e.*, DTIs) in ChEMBL v23. We applied several filtering and pre-processing steps to these data to create a reliable training dataset. First of all, data points were filtered with respect to “target type” (*i.e.*, single protein), “taxonomy” (*i.e.*, human and selected model organisms), “assay type” (*i.e.*, binding and functional assays) and “standard type” (*i.e.*, IC_50_, EC_50_, AC_50_, *K*_i_, *K*_d_ and Potency) attributes, which reduced the set to 3 919 275 data points. We observed that there were duplicate measurements inside this dataset that are coming from different bioassays (*i.e.*, 879 848 of the bioactivity data points belonged to 374 024 unique drug–target pairs). To handle these cases, we identified the median bioactivity value for each pair and assigned this value as the sole bioactivity measurement. At the end of this application, 3 413 451 bioactivity measurements were left. This dataset contained data points from both binding and functional assays. In order to further eliminate a potential ambiguity considering the physical binding of the compounds to their targets, we discarded the functional assays and kept the binding assays with an additional filtering on “assay type”. Finally, we removed the bioactivity measurements without a pChEMBL value, which is used to obtain comparable measures of half-maximal response on a negative logarithmic scale in ChEMBL. The presence of a pChEMBL value for a data point indicates that the corresponding record has been curated and, thus, reliable. After the abovementioned processing steps, the number of bioactivity points was 769 935.

Subsequently, we constructed positive (active) and negative (inactive) training datasets as follows: for each target, compounds with bioactivity values ≤10 μM were selected as positive training samples and compounds with bioactivity values ≥20 μM were selected as negative samples. In DEEPScreen, only the target proteins with at least 100 active ligands were modelled, in order to not lose the statistical power. This application provided models for 704 target proteins from multiple highly studied organisms. These organisms, together with the distribution of target proteins for each organism are as follows: *Homo sapiens* (human): 523, *Rattus norvegicus* (rat): 88, *Mus musculus* (mouse): 34, *Bos taurus* (bovine): 22, *Cavia porcellus* (guinea pig): 13, *Sus scrofa* (pig): 9, *Oryctolagus cuniculus* (rabbit): 5, *Canis familiaris* (dog): 3, *Equus caballus* (horse): 2, *Ovis aries* (sheep): 2, *Cricetulus griseus* (Chinese hamster): 1, *Mesocricetus auratus* (golden hamster): 1 and *Macaca mulatta* (rhesus macaque): 1. The UniProt accessions, encoding gene names, ChEMBL ids and taxonomic information of these proteins are given in the repository of DEEPScreen. Each target's training set contained a mixture of activity measurements with roughly comparable standard types (*e.g.*, IC_50_, EC_50_, AC_50_, *K*_i_, *K*_d_ and potency).

The selection procedure explained above generated positive and negative training datasets with varying sizes for each target. In order to balance the positive and negative datasets, we selected negative samples equal to the number of positive instances. However, for many targets, the number of negative points was lower than the positives. In these cases, we applied a target similarity-based inactive dataset enrichment method to populate the negative training sets (instead of randomly selecting compounds), using the idea that similar targets have similar actives and inactives. For this, we first calculated pairwise similarities between all target proteins within a BLAST search. For each target having an insufficient number of inactive compounds, we sorted all remaining target proteins with descending sequence similarity. Then, starting from the top of the list, we populated the inactive dataset of the corresponding target using the known inactive compounds of similar targets, until the active and inactive datasets are balanced. We applied 20% sequence similarity threshold, meaning that we did not consider the inactives of targets, whose sequence similarity to the query protein is less than 20%. The finalized training dataset for 704 target proteins contained 412 347 active data points (≤10 μM) and 377 090 inactive data points (≥20 μM). Before the negative dataset enrichment procedure, the total number of inactive instances for 704 targets was only 35 567. Both the pre-processed ChEMBL dataset (769 935 data points) and the finalized active and inactive training datasets for 704 targets are provided in the repository of DEEPScreen. We believe that the resulting bioactivity dataset is reliable and it can be used as standard training/test sets in future DTI prediction studies. The training data filtering and pre-processing operations are shown in Fig. 2.

### 4.2 Representation of input samples and the generation of feature vectors

In the DEEPScreen system, each compound is represented by a 200-by-200 pixel 2-D image displaying the molecular structure (*i.e.*, skeletal formula). Although 2-D compound images are readily available in different chemical and bioactivity databases, there is no standardization in terms of the representation of atoms/bonds, functional groups and stereochemistry. Due to this reason, we employed SMILES strings of compounds to generate the 2-D structural images, since SMILES is a standard representation that can be found in open access bioactivity data repositories, which contain the whole information required to generate the 2-D images. We employed the RDkit tool Python package (v2016.09.4) for image generation.^86^ A few examples from the generated images are shown in Fig. 1.

2-D images generated by RDkit are reported to have a standard and unique representation, which is achieved by applying a canonical orientation in all cases.^87^ There are special cases, which are not handled well, such as stereochemistry. However, this problem is not related to the generation of 2-D images by RDkit, but to the SMILES representations being non-stereospecific. In this study, we omitted stereochemistry since the cases correspond to an insignificant portion of the whole ChEMBL database.^88^

We carried out a small scale analysis to determine the input image size of the DEEPScreen system. We selected 100-by-100, 200-by-200 and 400-by-400 pixel image sizes for the test (sizes smaller than 100-by-100 were inadequate to draw molecules and sizes larger than 400-by-400 were too large to train the system with due to increased complexity). We generated the training and test compound images with the selected sizes for 3 target proteins: muscarinic acetylcholine receptor M5 (CHRM5) – CHEMBL2035, carbonic anhydrase VB (CA5B) – CHEMBL3969 and renin – CHEMBL286. After that, we trained 9 models (3 targets for 3 different images sizes) and optimized the hyper-parameters with grid-search. The finalized models were subjected to performance analysis by querying the test dataset compounds. We also recorded the average computational parameters in terms of run time and memory (the same amount of CPU power has been used for each model train/test run). The test results are given in ESI Table S2.† As shown in Table S2,† the average predictive performance (in terms of the MCC) significantly increased by 17% when the input image size is changed from 100-by-100 to 200-by-200. A similar performance increase was not observed when the input image size is changed from 200-by-200 to 400-by-400. Considering the run times, there was a significant increase both between 100-by-100 and 200-by-200, and 200-by-200 and 400-by-400. The run times for DCNN models were acceptable; however, it was not possible to train the Inception model with 400-by-400 due to extremely long run times. Considering the performance results along with the computational requirements, 400-by-400 was found to be non-feasible. Finally, for memory requirements, again the results were reasonable for DCNN models and for Inception models when the image sizes are either 100-by-100 or 200-by-200. These results indicated that the best performances were achieved with 200-by-200 image sizes, with reasonable computational requirements. As a result, 200-by-200 image size was chosen as default for the DEEPScreen system. Moreover, we observed in several cases that the size 100-by-100 was not sufficient to express large compounds properly. The whole image size analysis results are given in the repository of the study.

### 4.3 Neural network architecture of DEEPScreen

Deep convolutional neural networks are a specialized group of artificial neural networks consisting of alternating, convolution and pooling layers, which extracts features automatically.^89^,^90^ DCNNs have been dominating the image processing area in the last few years, achieving significantly higher performances compared to the state-of-the-art of the time.^89^,^91^,^92^ DCNNs run a small window over the input feature vector at both training and test phases as a feature detector and learn various features from the input regardless of their absolute position within the input feature vector. Convolution layers compute the dot product between the entries of the filter and the input, producing an activation map of that filter. Suppose that the size of the layer, on which the convolution operation is to be performed (layer #: *l* – 1) is *NxN* and the following convolutional layer has layer # *l*. Then, the value of the unit *x*_*ij*_ in the *l*^th^ layer, *xlij*, is calculated by the convolution operation (assuming no padding and stride of 1) using the following equation:1
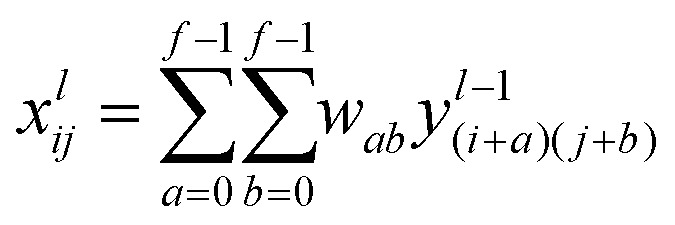



In the equation above, *f* stands for filter size, *w* stands for *fxf* filter and *yl*–1*ij*stands for the value of the *i*^th^ row and *j*^th^ column in the (*l* – 1)^th^ layer. Subsequently, a non-linear function *σ* such as the rectified linear unit (ReLU) is applied to *xlij*:

2 *ylij* = *σ*(*xlij*)

At the end of the convolution operation, the size of the *l*^th^ layer becomes (*N* – *f* + 1)*x*(*N* – *f* + 1). The parameters of the networks are optimized during the backpropagation step, by minimizing the following cross-entropy loss function:3
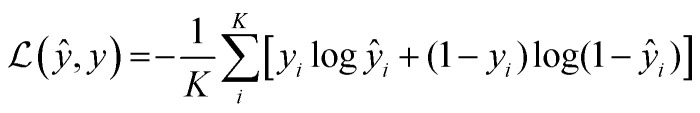



In the equation above, *ŷ* stands for prediction score, *y* stands for actual label and *K* stands for the number of examples in mini batches. Although the most standard form of DCNNs employ 2-D convolutions, 1-D or 3-D convolutions are possible.

Pooling layers combine the output of neuron clusters in one layer into a single neuron in the subsequent layer (*i.e.*, down-sampling) with the aim of reducing the number of parameters and the computational work and to prevent overfitting. A max pooling layer is commonly used in DCNNs and it works by running a window sequentially and taking the maximum of the region covered by the window, where each maximum value becomes a unit in the next layer. One of the most popular and widely used regularization techniques is dropout.^93^ This method randomly deactivates some of the neurons in various layers along with their connections at every epoch during the training procedure. By this, the system prevents overfitting; thus, the constructed models are more generalized.

In this study, we considered the DTI prediction as a binary classification problem, where the output can either be positive (*i.e.*, active, interacting or “1”) or negative (*i.e.*, inactive, non-interacting or “0”), referring to the relation between the query compound and the modelled target protein. For this purpose, an individual model was created for each target protein (*i.e.*, the single task approach). In terms of the employed DCNN architectures, we initially chose 3 options: Inception,^91^ AlexNET,^92^ and an in-house built DCNN architecture. The AlexNET architecture is a DCNN with stacked convolutional layers. It contains 5 convolutional and 3 fully connected layers. Inception is a highly specialized DCNN architecture. In standard DCNNs, filters with a uniform size are used in each level of convolutional layers, whereas in Inception, multiple filters with different sizes are combined in the same level (*i.e.*, Inception modules), to be able capture highly complex features. Various combinations of Inception modules are designed to create extremely deep and wide networks to achieve high predictive performance in practical training run times. Detailed information about the Inception network can be found in Szegedy *et al.*^91^ Both AlexNET and Inception displayed top performances in image classification tasks.^91^,^92^ For our in-house designed DCNN models, we used a simpler architecture (compared to Inception), which is composed of 5 convolutional + pooling and 1 fully connected layer preceding the output layer. Each convolutional layer was followed by a ReLU activation function and max pooling layers. The last convolutional layer is flattened and connected to a fully connected layer, followed by the output layer. We used the Softmax activation function in the output layer. A generic representation of the constructed DCNN models is given in Fig. 1. TFLearn framework version 0.3.2, cairosvg 2.1.2, and rdkit 2016.09.4 were employed for the construction of the DEEPScreen system.^94^

### 4.4 System training and test procedures

For each target protein model, 80% of the training samples (from both the positives and the negatives datasets) were randomly selected as the training/validation dataset and the remaining 20% was reserved for later use in the independent performance test procedure. Also, 80% of the training/validation dataset was employed for system training and 20% of this dataset was used for validation, during which the hyper-parameters of the models were optimized.

With the purpose of selecting the architecture(s) to be used in DEEPScreen, we initially trained and tested models for a small number of target proteins using a wide range of hyper-parameters. At the end of these initial tests, we eliminated the AlexNET architecture since its performance was inferior to the performances of the other two architectures. After this point, we continued our tests with Inception and our in-house DCNN architecture. We created and trained one model for each hyper-parameter selection, for each target, and for each architecture. The list of the hyper-parameters and the value selections are given in Table S1.† The models were run on the validation datasets during training to obtain the predictive performance (*i.e.*, accuracy, precision, recall, *F*1-score and MCC), which indicates the effectiveness of the pre-selected hyper-parameter values. At the end of the validation procedure, the best performing model (in terms of the MCC) was selected for each target. At the end of this analysis, our in-house DCNN architecture was selected for 397 of the target proteins and the Inception architecture was selected for the remaining 307 target proteins (out of the total of 704 targets). As a result, the finalized DEEPScreen system is composed of both Inception and in-house designed DCNN architectures. Next, test performances were calculated by running the finalized models on their corresponding independent test datasets, which have never been used before this point (*i.e.*, performances reported in the Results section). All of the training, test and prediction runs described in this study were carried out in parallel at the EMBL-EBI large-scale CPU cluster.

In order to investigate the possible reasons behind the performance differences between the Inception and the in-house DCNN architectures in DEEPScreen, we conducted a target protein family based comparison over our pre-trained 704 target protein models to observe if there is a performance difference between the two architectures for a specific protein family (*i.e.*, for how many members of a target protein family the Inception model was the best performer and for how many of them the in-house DCNN was the best). We found out that the architectures performed nearly the same for nuclear receptors. Considering the rest of the families, the DCNN architecture performed better between 28% and 50%, compared to the Inception models. We believe the only reason behind observing this performance difference is that the Inception architecture is significantly more complex and computationally more demanding compared to the in-house DCNN architecture, and as a result, the hyper-parameter space that we were able to scan during the grid search analysis was smaller for Inception. The computational requirement difference between Inception and the in-house DCNN is also given in ESI Table S2,† calculated for three target proteins. A grid search with the same hyper-parameter space size for Inception models would probably result in predictive performances greater than or equal to the performance of the DCNN models. However, a grid search of this magnitude would require a very long time to finish even on a strong computing resource. To test this idea, we analyzed the Inception and in-house DCNN performances over 114 target proteins, all of which were difficult to model, as pointed out by the low predictive performances in our initial tests. For these 114 targets, we trained our predictive models and searched large hyper-parameter spaces for both the Inception and in-house DCNN models, and selected the best Inception and the best in-house DCNN for each of the 114 targets by checking the highest test performance in terms of the MCC measure. After that, we compared the best Inception model with the best in-house DCNN model, for each target (*i.e.*, 114 comparisons in total). We found that in-house DCNN models performed better for 42 of the targets and the Inception model performed better for 35 of them (the performance was exactly the same for the remaining 37 targets). We also calculated the actual performance differences between the best in-house DCNN and the best Inception models for each target, and found that the average performance difference was the same when we compared two groups: (1) targets on which the DCNN performed better and (2) targets on which Inception performed better. These results indicated that there is no significant performance difference between Inception and the in-house DCNN, when similar hyper-parameter spaces are searched during the model optimization step. The results of the Inception *vs.* in-house DCNN performance analysis have been provided in the repository of the study.

### 4.5 Benchmark datasets for the predictive performance comparison

All of the four non-random split datasets used in the performance analyses are constructed by considering scaffold/structure/temporal train-test sample divisions; as a result, they accurately simulate real-case prediction scenarios, where the predictive systems are queried with completely new compounds with different features (*e.g.*, never-seen-before scaffolds).

First of all, we aimed to generate our own bias free benchmark dataset using our fundamental ChEMBL training set. For this, we first focused on further eliminating the negative selection bias, even though we previously showed that similarity among negative samples was around the same level as the similarity between negative (inactive) samples, in our fundamental datasets (please see the Results section), mainly due to the fact that we only included compounds with real experimental bioactivity measurements (coming from binding assays) against the intended target. For further elimination of negative selection bias, we identified the negative dataset compounds, whose all activity data points (against all targets) in the ChEMBL database are in the inactives range (*i.e.*, ≥20 μM ×C50) and discarded them. The compounds which have at least one data point in the actives range (for any target) were kept in the negative datasets. Considering the rigorous filtering operations applied to generate our source/fundamental bioactivity dataset (explained in the Methods section in detail), we assumed that even one active data point (*i.e.*, ≤10 μM ×C50) would be sufficient to accept that the corresponding molecule does not possess features that make it an all-inactive/invalid compound. To eliminate chemical bias from our datasets, we applied the Murcko scaffold^46^ detection and train-test split (based on the detected scaffolds) module in the RDKit package. This way, for each target, all compounds with a distinct scaffold either ended up in the training set or in the test set; in other words, the compounds with the same scaffold were not distributed to both training and test. Following these rules, we carefully constructed train and test datasets for 17 representative targets spanning the main target families of enzymes, GPCRs, ion channels, nuclear receptors and others, with dataset sizes ranging from 143 to 5229. The total number of data points in the finalized dataset was 21 200. The targets were selected mostly based on the representative drug targets list given in another study.^44^ We selected 10 targets from the list given by Mysinger *et al.* (many of the remaining targets listed in this article were not among the 704 DEEPScreen targets, so they could not be covered); we additionally included renin and JAK1 (since these two targets were also selected as use cases for further validation) and 5 additional randomly selected targets proteins (from different families), to reflect the target protein family distribution for 704 DEEPScreen targets. The gene names of the selected 17 targets are MAPK14, JAK1, REN, DPP4, LTA4H, CYP3A4, CAMK2D, ADORA2A, ADRB1, NPY2R, CXCR4, KCNA5, GRIK1, ESR1, RARB, XIAP, and NET, summing into 7 enzymes (taking the distribution of the enzyme sub-families into account as well), 4 GPCRs, 2 ion channels, 2 nuclear receptors and 2 others. We named this set the representative target benchmark dataset.

The second benchmark dataset we used in our study was directly obtained from the study by Lenselink *et al.*^18^ In this study, the authors created a high quality ChEMBL (v20) bioactivity dataset that includes 314 767 bioactivity measurements corresponding to target proteins with at least 30 bioactivity data points. They used pChEMBL = 6.5 (roughly 300 nM) bioactivity value threshold to create active and inactive compound datasets for each target. The authors evaluated their method with a test dataset created by a temporal split, where for each target protein, all of the bioactivity data points reported in the literature prior to 2013 were used in the training, and the newer data points were gathered for the test dataset. This test dataset is more challenging for ML classifiers compared to any random-split dataset.

The third dataset we used was Maximum Unbiased Validation (MUV), another widely-used benchmark set, composed of active and inactive (decoy) compounds for 17 targets.^95^ The MUV dataset was generated from the PubChem Bioassay database. The active compounds in this dataset were selected to be structurally different from each other. Therefore, it is a challenging benchmark dataset, which avoids the bias rooting from highly similar compounds ending up in both training and test splits (*i.e.*, chemical bias). There are 17 targets in the MUV dataset, together with 30 actives and 15 000 decoys for each target.

The fourth benchmarking dataset employed in this study was DUD-E, a well-known set for DTI prediction, which includes curated active and inactive compounds for 102 targets. The active compounds for each target were selected by first clustering all active compounds based on the scaffold similarity and selecting representative actives from each cluster. The inactive compounds were selected to be similar to the active compounds in terms of the physicochemical descriptors, but dissimilar considering the 2-D fingerprints.^44^ The benchmark dataset consists of 102 targets, 22 886 actives (an average of 224 actives per target) and 50 property-matched decoys for each active, which were obtained from the ZINC database.^44^ It is also important to note that the DUD-E benchmark dataset is reported to suffer from negative selection bias problem; as a result, we did not conclude our results on the performance on the DUD-E dataset. We just used the DUD-E dataset to make a highly generic performance comparison with the literature, since DUD-E is a widely used benchmark dataset.

### 4.6 *In vitro* validation of JAK signalling as a new target for cladribine

Cytotoxicity assays were performed on well differentiated Huh7 (2500) and HepG2 (3000) and poorly differentiated Mahlavu (1000) primary liver cancer cells that were plated and cultured in 96-well cell culture plates in Dulbecco's modified Eagle medium (DMEM) supplemented with 10% fetal bovine serum (Gibco, Invitrogen), 1% non-essential amino acids (Gibco, Invitrogen) and 100 units per ml penicillin/streptomycin (Gibco, Invitrogen) at 37 °C under 5% CO_2_ for 24 hours. Cells were then treated with 2-chloro-2′-deoxyadenosine (cladribine) (Santa Cruz, # sc-202399) in DMSO (Sigma) in a concentration gradient (40, 20, 10, 5, 2.5, 1.25, 0.625, and 0.3 μM in triplicate). At 72 hours, NCI-SRB assay was performed and IC_50_ values were calculated (*R*^2^ ≥ 0.9) (Table S3†).^93^ JAK downstream effector STAT3 associated cellular phosphorylation alteration was assessed by flow cytometry. Huh7 (500.000), Mahlavu (250.000), and HepG2 (750.000) cells were grown in 10 cm plates for 24 hours, and then Huh7 and Mahlavu cells were treated for 72 hours with 1 μM and HepG2 cells were treated with 3 μM cladribine. Cells were then collected and stained with Alexa Fluor® 647 Anti-STAT3(p-Y607) (BD Biosciences, #557815) according to the manufacturer's protocol and were analysed using an ACEA Novocyte flow cytometer.

Huh7 (500.000), Mahlavu (250.000), and HepG2 (750.000) cells were plated in 100 mm culture dishes for 24 hours. HepG2 and Mahlavu cells were then treated with 2 μM or 1 μM and Huh7 cells were treated with 6 μM or 3 μM Huh7 cladribine for 48 or 72 hours. After 48 or 72 hours of incubation, cells were fixed with ice-cold 70% ethanol for 3 hours at –20 °C. Cell cycle analysis was then carried out with a PI (propidium iodide) Muse™ cell cycle kit (Millipore, MCH1000106) and apoptosis was demonstrated with annexin V assay (Roche, #11 858 777 001) by flow cytometry. Cellular STAT3 and p-STAT3 protein levels were examined on western blot using STAT3 (CST #9139S) and phospho-STAT3 (CST, #9145S) antibodies. Calnexin (CST, #2679) was used for equal loading control. Proteins were visualized using an Odyssey CLx-LICOR imaging system. DMSO was used as the vehicle control in all experiments.

### 4.7 Literature based validation of novel DTI predictions

DEEPScreen produced 21.2 million completely novel DTI predictions. As a result, it was not possible to manually check the literature if a research group has already studied these specific drug/compound-target interactions for validation. Instead we assumed a more directed approach, where the validation cases were determined from a newer version of ChEMBL and from the literature first, and then, DEEPScreen's novel prediction results were searched to observe if these interactions were identified by DEEPScreen as well. The selected cases are composed of two types of data points. The first one concerns the already approved drugs (or the ones in the experimental development phases), where the given target interactions are novel (*i.e.*, not part of the already approved or experimental treatment for these drugs) and thus, serve the purposes of drug repositioning. For this, we found the cases where the corresponding drug has bioactivity data points for new targets in ChEMBL v24, which were not part of v23 (ChEMBL v23 was used for the training of DEEPScreen). As such, these cases correspond to the recently curated data. Using this set, we only selected the cases where the corresponding targets were among the 704 target proteins of DEEPScreen, and the source publications of the reported bioactivities were novel (*i.e.*, from 2016 and 2017). It was not possible to find any case with 2018 publications since these articles are not curated in ChEMBL yet. We then searched DEEPScreen large-scale prediction results to find if these cases were predicted. The results only display a few of the coinciding data points with the most novel source publications. The second type of data points consists of completely novel bio-interactions that have not entered ChEMBL or any other bioactivity database yet. Since these compounds are not incorporated into ChEMBL, our large-scale prediction results did not include them. To observe if DEEPScreen can predict the reported activities given in 2 selected drug design and development publications from 2018,^84^,^85^ we generated the SMILES representations and the 2-D structural images of the documented compounds using their molecular formula as reported in the corresponding publications. After that, we run the query compounds against their newly identified targets (which were reported in the respective articles) to see if DEEPScreen can predict these highly novel interactions. For the literature-based validation analysis, the approved and experimental drug information was obtained from the DrugBank database.^6^

### 4.8 Molecular docking experiments

For renin docking experiments, the crystal complex structure of human renin protein, bound to its approved drug aliskiren, was employed (PDB id: 2V0Z). To prepare the structure for docking, first of all, the O chain was deleted from the 2-chain homodimer structure (only the C chain was kept) since the known binding pocket lies within each chain and not on the interface between the chains. Second, all of the ligand atoms and water molecules have been removed, except two water molecules that were reported to mediate the hydrogen bonding with aliskiren (#184 and 250).^60^,^96^ The modified protein structure was given as input to the MTiAutoDock service^97^ together with the sdf format ligand structure files (*i.e.*, aliskiren, remikiren, cortivazol, misoprostol, lasofoxifene, sulprostone, acetylsalicylic acid, calcifediol, difluprednate and mivacurium) obtained from the ZiNC (v15) database.^98^ A binding pocket was also created at the input level using the known binding sites in the crystal structure;^99^ this way, all molecules were docked into the corresponding pocket. MTiAutoDock service has automatically added the hydrogen atoms to the crystal structure and executed the docking procedure using AutoDock 4.2.6.^100^ We also replicated the exact same experiment using the SwissDock web service.^101^ We used the same processed pdb file for the receptor structure and employed ligand structures in mol2 format, downloaded from the ZiNC database. We defined the region of interest for the local docking by calculating the mean coordinates of the reported interacting atoms (*x*: 11.04, *y*: 46.86, *z*: 69.53) in the renin–aliskiren complex structure (PDB id: ; 2V0Z) and we defined a grid size of 20 × 20 × 20 Å. Hydrogen atoms and missing side chains were automatically added to the structure. For both MTiAutoDock and SwissDock dockings, the best poses were evaluated *via* binding free energy calculations and the one with the lowest energy was selected as the finalized result in each docking run.

For the RXRbeta docking experiment, the crystal complex structure of human LXRalfa-RXRbeta ligand-binding domain heterodimer, bound to metoprenic acid, was used (PDB id: 1UHL). In order to prepare the structure for docking, chain A was extracted from the PDB file. The tretinoin (*i.e.*, all-trans retinoic acid) molecule file was downloaded from the ZiNC (v15) database in mol2 and sdf formats (id: ZINC12358651). The applied docking procedure was the same as that described above for renin dockings. UCSF Chimera (v.1.13.1) software^102^ was used for the visualization of docking results.

### 4.9 Performance evaluation metrics

We mainly used 3 evaluation metrics, *F*1-score, Matthews correlation coefficient (MCC) and area under receiver operating characteristic curve (AUROC), to evaluate the predictive performance of DEEPScreen and to compare its results with other DTI prediction methods. The formulae of these evaluation metrics are given below together with precision and recall that make up the *F*1-score:4
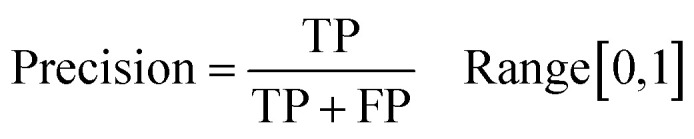

5
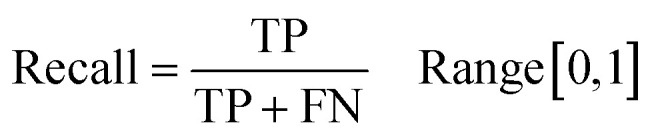

6
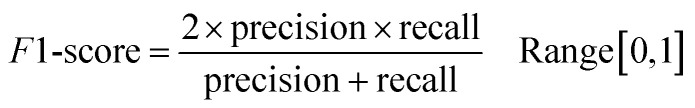

7
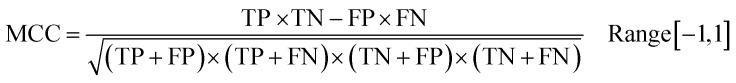



In the equations above, TP (*i.e.*, true positive) represents the number of correctly predicted interacting drug/compound-target pairs and FN (*i.e.*, false negative) represents the number of interacting drug/compound-target pairs that are predicted as non-interacting (*i.e.*, inactive). TN (*i.e.*, true negative) denotes the number of correctly predicted non-interacting drug/compound-target pairs, whereas FP (*i.e.*, false positive) represents the number of non-interacting drug/compound-target pairs, which are predicted as interacting.

## 5. Data and code availability

The source code, all datasets and the results of this study are available at: https://github.com/cansyl/DEEPscreen.

## 6. Author contributions

ASR, VA, MJM, RCA, and TD conceived the idea. ASR, VA and TD performed DEEPScreen system construction. ASR coded the system and prepared the datasets. ASR and TD performed the data analysis including the statistical tests. TD carried out the molecular docking experiments. ES and RCA performed the wet-lab experiments. ASR, ES, VA, RCA and TD wrote the manuscript. MJM, RCA, VA and TD supervised the overall study. All authors have revised and approved the manuscript.

## Conflicts of interest

There are no conflicts to declare.

## Supplementary Material

Supplementary information
