# Supplementary material for: DEEPScreen: high performance drug–target interaction prediction with convolutional neural networks using 2-D structural compound representations
Source: Chem Sci. 2020 Jan 8;11(9):2531–57. doi: 10.1039/c9sc03414e (PMC7643205; doi:10.1039/c9sc03414e)
Supplement: Supplementary file 1 [file SC-011-C9SC03414E-s001.pdf]

Electronic Supplementary Material (ESI) for Chemical Science.
This journal is © The Royal Society of Chemistry 2020

Supplementary Material

DEEPScreen: High Performance Drug-Target Interaction Prediction with Convolutional Neural Networks Using 2-D Structural Compound Representations

Ahmet Sureyya Rifaioglu[1,2,3], Esra Nalbat[3], Volkan Atalay[1,3,*], Maria Jesus Martin[4], Rengul Cetin-Atalay[3,5], Tunca Doğan[6,7,*]

[1] Department of Computer Engineering, METU, Ankara, 06800, Turkey

[2] Department of Computer Engineering, İskenderun Technical University, Hatay, 31200, Turkey

[3] KanSiL, Department of Health Informatics, Graduate School of Informatics, METU, Ankara, 06800, Turkey

[4] European Molecular Biology Laboratory, European Bioinformatics Institute (EMBL-EBI), Hinxton, Cambridge, CB10 1SD, UK

[5] Section of Pulmonary and Critical Care Medicine, the University of Chicago, Chicago IL 60637, USA.

[6] Department of Computer Engineering, Hacettepe University, Ankara, 06800, Turkey

[7] Institute of Informatics, Hacettepe University, Ankara, 06800, Turkey

* To whom correspondence should be addressed. Tel: +905055250011; Email: tuncadogan@hacettepe.edu.tr
Correspondence may also be addressed to. Tel: +903122105576; Email: vatalay@metu.edu.tr

1. Introduction

1.1 Drug-Target Interaction Prediction

Starting from the 2000s, developing computational methods to aid the drug discovery process by predicting the unknown interactions between drugs / drug candidate compounds and target biomolecules (i.e., drug target interaction -DTI- prediction or virtual screening) [1,2] started to become a

main-stream research area. Most of the DTI prediction methodologies are based on the idea that similar structures have similar activities [3,4] and utilize the experimentally identified drug-target interaction information coming from bioassay results for learning. DTI prediction methods usually employ supervised machine learning (ML) models to find new compounds (or targets) that possess features similar to the known drugs (or targets) [2,5,6].

1.2 Feature Engineering in DTI Prediction

One of the essential steps in ML method development is feature engineering, which constitutes designing, pre-processing and extracting meaningful features to be used for system training. In ML-based DTI prediction, feature vectors correspond to fixed-dimensional quantitative representations/descriptors of the input samples (i.e., drugs and/or targets), used to characterize the molecular properties that play role in the interactions, so that the ML algorithm can learn from these features to accurately predict unknown DTIs. In computational drug discovery studies, feature engineering is generally performed using computationally intensive third party methods/tools, where the main limitation is the constructed features not generalizing well to the whole proteochemical space [7], also, they often suffer from the curse of dimensionality [8]. Numerous different types of compound and protein descriptors have been employed for the generation of feature vectors in DTI prediction so far [2,9], though benchmarking studies have indicated that there is no consensus on what are the sole best compound and target protein descriptors [10,11]. On the compound side, bit-string based compound descriptors (fingerprints), binary feature vectors where each dimension represents the presence or absence of sub-structures on a compound, are widely used. Extended Connectivity Fingerprints - ECFPs [12] are one of the most widely used compound descriptors and represents the current state-of-the-art. The main problem related to the employment of fingerprints for DTI prediction is that, they represent only a pre-selected set of chemical sub-structures that are known to play roles in the interaction with target proteins [13]. The rest of the structural aspects are omitted from these representations. However, there are less explored parts of the DTI space, where the important sub-structures are not clearly known. As a result, it is debated that bit-string based descriptors have issues related to generalization, considering their power of representation of drug-target relations,

over many different families of proteins and compounds [13,14]. To overcome this problem, fingerprints with larger bit space were proposed [11,15]; however, the ones that represent a large portion of the molecular properties of compounds are usually extremely high dimensional and impractical to be used in a ML-based predictive method due to the computational burden and the bear risk of falling into the "curse of dimensionality".

With the aim of developing DTI prediction methods, a diverse set of ML techniques are employed (together with the feature vectors generated using abovementioned descriptors) such as random forest (RF) [16,17], support vectors machines (SVM) [17,18], and logistic regression (LR) [19].

1.3 Deep Learning Applications in Drug Discovery

The term "deep learning" (DL) is coined for the novel neural network architectures that perform significantly better compared to conventional classifiers especially in the fields of computer vision and natural language processing, mainly due to multiple layers of data abstraction [20]. Deep neural networks (DNN), a sub-group of DL techniques, are artificial neural networks with high complexity, composed of multiple hidden layers [21]. Lately, DL-based classifiers are also started to be applied for DTI prediction. In one of the earliest applications, Ma *et al.* constructed feed-forward DNN Models using molecular compound descriptors to predict diverse interactions in Merck's QSAR challenge data sets, and showed that DNNs perform better compared to conventional ML techniques[22]. Lenselink *et al.* proposed a proteochemometric modelling (PCM) based method for DTI prediction, where both compound and target features were employed (i.e., molecular fingerprints for compounds and a custom built composite descriptor -mainly including physicochemical properties- for targets as described by van Westen *et al.*[10]) as 1-D vectors for the training within a multi-layered perceptron DNN architecture [23]. AtomNet, a structure-based virtual screening method, uses convolutional neural networks (CNNs) for DTI prediction. This method incorporates 3-D structural features of known compound-target complexes to model DTIs [24]. Ragoza *et al.* proposed convolutional neural network based scoring function approaches for ranking and predicting binding affinities and poses in the process of structure-based drug design, which outperformed widely used the AutoDock Vina scoring function [25]. Gonczarek *et al.* developed a method that uses specific binding pockets of targets along

with fingerprints extracted using the 3-D structural features of compounds [26]. Altae-tran *et al.* proposed a DL-based method called "iterative refinement long short-term memory" using graph convolutions, where the input of the system is the 2-D graph structure of compounds [27]. They employed one-shot learning methodology, where the aim is to create predictors for the targets having low number of training samples. Kearnes *et al.* employed graph convolutions to learn features using graph structures of compounds [13]. The field of the DL-based DTI prediction is still in its infancy and the studies published so far were mostly focused on the applicability of DL algorithms and prototyping [13,22,23,26,28,29]. The results of these studies have indicated that DL has a great potential to advance the field by identifying unknown DTIs at large-scale [13,23,24,26–30]. Apart from the high predictive performance, another advantage of employing DL-based DTI predictors is the minimal requirement of feature engineering as these algorithms are able to extract complex and meaningful features from the raw data, automatically [31].

2. Results

2.1 Protein Family-Based Analysis of DEEPScreen Targets

In order to carry out a global structural and functional analysis on DEEPScreen's 704 target proteins, we extracted statistics in terms of the distribution of the number of structural domains they contain (from InterPro [32] and UniProt-DAAC [33]), their sequence length/sizes (from UniProtKB [34]), total number of family/domain/repeat-motif annotations (from InterPro), number of Gene Ontology based functional annotations (from UniProt-GOA), and the number of experimentally identified active ligands (from ChEMBL), based on the high level families of these proteins (i.e., enzymes, GPCRs, nuclear receptors, ion channels and others). We also examined the existence of 3-D structural information (from PDB), active and binding site annotations (from UniProtKB) and disease indications (from UniProtKB [34], OMIM [35] and OrphaNet [36]) on these target proteins. Figure 3 displays the results using percentage-based histogram and bar plots (i.e., the sum of all bars for each protein family gives 100%). Figure 3.a shows the histograms for the number distribution along with the protein family size pie chart. Figure 3.b displays the bar graphs indicating the difference between

existence and non-existence of a particular type of annotation among the members of each target protein family. As observed from Figure 3.a, target protein sequence lengths and the number of domains they contain are highly variable between protein families. GPCRs are generally shorter and contain fewer domains (usually only 1) compared to the remaining of the families. In terms of the number of family/domain/repeat-motif and GO-based function annotations, there is no significant difference between the target protein families and the majority of all targets are well annotated. Considering the number of known interacting compounds (i.e., ligands) from ChEMBL, GPCRs rank the first; enzymes, nuclear receptors and others families have similar number of known ligands and rank second, whereas ion channels slightly falls behind. It is important to note that, this analysis has been done over the 704 target proteins of DEEPScreen, each of which has at least 100 known ligands, as a result, the statistics given here does not reflect the whole target protein space. As observed from Figure 3.b, 80% of the targets in the enzymes family have at least one 3-D structural entry (in PDB) associated to it. This is 100% for nuclear receptors, however only 33% for GPCRs. The rightmost bar in each bar plot shows the percentage considering all of the 704 targets. In terms of disease indications associated with target proteins, family-based differences are mostly insignificant, with nuclear receptors having the highest rate (60%). For binding and active site annotations, enzymes ranked first with a significant margin, "others" family came second with 35 and 15 targets (out of 58) containing at least one binding and active site annotation, respectively. GPCRs, ion channels and nuclear receptors have no active site annotation since this property is mostly associated with enzymatic activities. Overall, all of these proteins have high amount of annotations in the biological databases, since they are highly studied in the biomedical research field in terms of their high potential to be parts of novel treatment options.

2.2 System Robustness Against Input Image Transformations

One of the critical points in the computer vision tasks is the system robustness concerning the differences in the representations of the object of interest, such as the viewing angle or the scale. In DEEPScreen, input images are standardized by computationally generating them from SMILES representations, this way all images have similar representations in terms of viewing angle (i.e.,

rotation). However, we investigated the question of how the models would behave if they are provided with rotated compound images as the query. For this, we have selected 3 target protein models (i.e., BCHE gene - CHEMBL5077, GSK3beta gene - CHEMBL3638364, PTGS1 gene - CHEMBL221) and we constructed the rotated compound images of the positive and negative performance test dataset compounds of these targets. 7 new samples were generated from each test compound image, by rotating the original image by 45 degree angle. We fed these rotated images to the original pre-trained predictive models as the query set. Since the original models have never seen these rotated images before (during training), the performances were decreased by 29%, 23% and 32% in terms of the MCC measure and by 22%, 18% and 18% in terms of accuracy for BCHE, GSK3beta and PTGS1 models, respectively (compared to the original test performances of these models, when there is no rotated images in the query set). It was argued in the literature that the application of training data augmentation by generating and adding new samples to the training set by rotating the existing images solves this problem [37]. To observe if this is the case for DEEPScreen, we generated rotated compound images for each and every positive and negative training dataset instance (using the same 45 degree rotation approach), and re-trained the same predictive models using the enhanced training datasets and performed the hyper-parameter optimization tests with grid-search. After that, we measured the performance of these newly trained models by querying them with the rotation-added test datasets used in the previous test. Finally, we compared the performance of the rotation-trained models with the performance of the original models (i.e., models without any rotated images in training or in test datasets). The results showed that, when the rotated images were added to the training, the performance remained roughly the same for all 3 selected models (i.e., 0-2% performance decrease in both MCC and accuracy for BCHE, GSK3beta and PTGS1), which indicates that training with data augmentation by generating rotated data points worked well. However, it was not possible to apply this methodology to train all of 704 target protein models of DEEPScreen due to significantly increased computational complexity. The application of rotated compound images increased the training dataset size of each model 8 times relative to the original training datasets. This was a huge burden especially for the complex Inception architecture-based models. Considering the fact that whole hyper-parameter optimization by grid search procedure should have been repeated, it

was not possible to construct a rotation invariant system for DEEPScreen in the end. Instead, we relied on generating canonical images for both the training and query compounds from SMILES as the input, which worked well in practice. There are also novel alternative solutions proposed lately in the literature such as the approach proposed by Thomas *et al.*, where the authors developed a DCNN architecture that is equivariant to rotations by using filters from spherical harmonics [38]. Similar approaches may be applied in the future to modify DEEPScreen to make it completely rotation invariant so that the user drawn images can directly be fed to the system as the input (through a web-interface) instead of SMILES.

Another important transformation type for computer vision tasks is the scaling. In DEEPScreen, the compounds are drawn as images by fully occupying the 200-by-200 pixel frame no matter what their actual molecular sizes are. This means that a certain component (i.e., a sub-structure such as a benzene ring) in a large (i.e., high molecular weight) compound will occupy fewer number of pixels (i.e., appear smaller in the image) compared to the size of the same sub-structure as a part of a smaller (i.e., low molecular weight) compound. The predictive system should be invariant to these scaling variances, in other words, it should perceive, for example, a benzene ring structure independent from its size on the compound images. This is generally achieved by training CNN-based systems by using input instances containing the features of interest in different scales [37]. In DEEPScreen, this is automatically achieved since the training dataset compounds of each target protein contain both relatively larger and smaller molecules. In order to examine this issue, we conducted a scaling analysis on our case study model: renin. In this analysis, we augmented the test dataset of renin target protein model by scaling both the positive and negative test dataset molecules by decreasing the sizes by 10%, 20% and 30% (i.e., molecules occupy a smaller area on the 200-by-200 pixel images). In the end, we obtained a test dataset 4 times larger compared to the original set (i.e., original + 10% + 20% + 30% scaled down compounds for all test samples). We fed the newly generated scaled test dataset as query to the original pre-trained predictive model of renin target protein. The assumption here was that, since the training dataset compounds of renin contained samples in different scales (i.e., same sub-structures automatically drawn in different sizes in different images due their presence in both

large and small compounds) the system will be robust against the variance in the artificially scaled samples in the test dataset. The performance results of this test have indicated that decreasing the molecule size by 10% affected the predictive performance by reducing precision, recall, F1-score and accuracy by 9%, 7%, 8% and 9%, respectively. A same trend was observed for 20% and 30% size reductions (i.e., 8-10% performance reduction compared to the test results of the previous scaling), as well, pointing out a linear relation between molecule size scales and the performance. The results pointed out that the performance change observed with a 10% scaling is acceptable.

Nevertheless, it is possible to query a target protein model with a compound that is significantly larger or smaller compared to all training dataset samples of the model. In this case, the system may misinterpret a feature/sub-structure since it will be scaled very differently compared to the scales it is aware of (from the training data). However, a molecule that is significantly larger or smaller compared to all known ligands of a target would be less likely to interact with the intended target due to its inability to occupy the intended binding region/pocket. In order to test this, we analyzed the size distribution of the compounds in the active and inactive training datasets of renin, in terms of molecular weights, since the molecular weight can be a good indicator of the scale of the compounds on the 2-D images. The molecular weight is only consist of a number (i.e., we can compare different molecular weights on a 1 dimensional space), whereas in images, compounds are represented over 2 dimensions (where there is more space), as a result, 2-D scaling differences are roughly equivalent to the square of the molecular weight differences (e.g., a 10% size difference in 2-D images is roughly equivalent to 31.5% difference in molecular weight). We also manually checked several compound images from the original training datasets of different target proteins and observed that the abovementioned relation between the molecular weight change and 2-D scaling change holds true.

The analysis of the training dataset compounds of an example target protein: "Complement factor D" (gene name: CFD, ChEMBL id: CHEMBL2176771), which has 732 active ligands (close to the average number of active ligands for all DEEPScreen targets: 728), revealed that the molecular weight distribution is normal and the active compounds have a mean of 504 g/mol and a standard deviation of 38 g/mol, which indicates a weight change of 26% between +/- two standard deviations

from the mean (i.e., 580 to 428 g/mol), translating into roughly 7% (i.e., 0.26^2) scaling difference on 2-D. It is important to note that two standard deviations from the mean covers nearly 95% of all data points in a normal distribution. This places the expected scaling difference between the potential ligands of a target within an acceptable zone in terms of model performance reduction (i.e., < 10%).

3. Discussion

In DEEPScreen, we selected a considerably loose threshold (i.e., 10 μM) bioactivity value to label training instances as active (i.e., interacting), which in turn resulted in high number of predictions. Considering an accurate "active" prediction, the true experimental bioactivity measurement can go as high as 10 μM (IC50), which may be unacceptably high especially for certain target families (e.g., kinases and GPCRs). However, our aim in developing DEEPScreen was to aid experimental researchers in drug discovery and repurposing by providing all novel matches that would potentially raise an interest. In this sense, we believe that the results of our large-scale DTI production run will be useful.

The selected values for the hyper-parameter optimization tests are given in Table S.1. The determination of the hyper-parameter value alternatives was made according to previous literature on DL-based method development. It was not possible to carry out an exhaustive hyper-parameter optimization methodology, such as a full grid search, during the development of DEEPScreen, due to high computational burden (especially for the Inception architecture). Instead, manual selections were made to obtain satisfactory results by practically scanning a portion of the whole hyper-parameter space. It might be possible to further increase the predictive performances of our target-based models by scanning an even more diverse set of hyper-parameters; however, the computational cost was already extremely high. As a result, we decided to stop the process after achieving satisfactory results.

Deep neural networks have long been criticised about being black-boxes, where it is not possible to observe why a system behaves the way it does. This has been especially valid in the field of ML applications to biological data. On the other hand, there has been a recent focus in the DL literature on dissecting the trained models to understand what piece of information has been extracted/learnt at

each block of the model. This also gave birth the idea of re-using the relevant blocks inside different models, for relevant predictive modelling purposes. In this context, it would be interesting to observe the extracted features (i.e., patterns) from the compound images as it is highly probable that most of these features are important to the task at hand (i.e., physical interactions with proteins). For example, they may correspond to the binding regions of compounds. An analysis in this sense can reveal information about the unknown binding properties, which in turn can be used in *de novo* drug design.

4. Conclusions

In this work, we have shown that deep convolutional neural networks can be utilized to successfully predict the drug-target interactions, using only the 2-D structural images of drugs and drug candidate compounds. The proposed method, DEEPScreen, has been tested on various benchmarking datasets and compared with both the state-of-the-art and with highly novel DTI prediction methodologies to reveal that it performs well. The large-scale DTI prediction run that virtually scanned more than 1 million compounds against the modelled target proteins has produced 21.2 million completely novel data points, which can be utilized by the experimental and computational researchers to aid their work on drug design and repurposing, as an early selection of candidate compounds or scaffolds. We investigated several examples in the manuscript regarding the relevance of the prediction results to be used towards drug repurposing. We also constructed DEEPScreen as a collection of ready to use predictive models so that a newly synthesized (or *in silico*) molecule can easily be tested against 704 target proteins that DEEPScreen has predictive models for.

5. References

(1) Shoichet, B. K. Virtual Screening of Chemical Libraries. *Nature* **2004**, *432* (7019), 862–865. https://doi.org/10.1038/nature03197.

(2) Rifaioglu, A. S.; Atas, H.; Martin, M. J.; Cetin-Atalay, R.; Atalay, V.; Doğan, T. Recent Applications of Deep Learning and Machine Intelligence on in Silico Drug Discovery: Methods, Tools and Databases. *Brief. Bioinform.* **2018**, No. September, 1–36. https://doi.org/10.1093/bib/bby061.

(3) Martin, Y. C.; Kofron, J. L.; Traphagen, L. M. Do Structurally Similar Molecules Have Similar Biological Activity? *J. Med. Chem.* **2002**, *45* (19), 4350–4358. https://doi.org/10.1021/jm020155c.

(4) Bender, A.; Glen, R. C. Molecular Similarity: A Key Technique in Molecular Informatics. *Org. Biomol. Chem.* **2004**, *2* (22), 3204–3218. https://doi.org/10.1039/b409813g.

(5) Lavecchia, A. Machine-Learning Approaches in Drug Discovery: Methods and Applications. *Drug Discovery Today*. 2015. https://doi.org/10.1016/j.drudis.2014.10.012.

(6) Chen, H.; Engkvist, O.; Wang, Y.; Olivecrona, M.; Blaschke, T. The Rise of Deep Learning in Drug Discovery. *Drug Discov. Today* **2018**, *23* (6), 1241–1250. https://doi.org/10.1016/j.drudis.2018.01.039.

(7) Pahikkala, T.; Airola, A.; Pietila, S. Toward More Realistic Drug-Target Interaction Predictions. *Brief. Bioinform.* **2014**, *16* (2), 325–337. https://doi.org/10.1093/bib/bbu010.

(8) Searls, D. B. Data Integration: Challenges for Drug Discovery. *Nat. Rev. Drug Discov.* **2005**, *4* (1), 45–58. https://doi.org/10.1038/nrd1608.

(9) Todeschini, R.; Consonni, V. *Molecular Descriptors for Chemoinformatics, Volumes I & II*, 2nd ed.; Wiley-VCH, 2009.

(10) Van Westen, G. J. P.; Swier, R. F.; Cortes-Ciriano, I.; Wegner, J. K.; Overington, J. P.; Jzerman, A. P. I.; Van Vlijmen, H. W. T.; Bender, A. Benchmarking of Protein Descriptor Sets

in Proteochemometric Modeling (Part 1): Comparative Study of 13 Amino Acid Descriptor Sets. *J. Cheminform.* **2013**, *5* (41), 1–11. https://doi.org/10.1186/1758-2946-5-42.

(11) Sawada, R.; Kotera, M.; Yamanishi, Y. Benchmarking a Wide Range of Chemical Descriptors for Drug-Target Interaction Prediction Using a Chemogenomic Approach. *Mol. Inform.* **2014**, *33* (11–12), 719–731. https://doi.org/10.1002/minf.201400066.

(12) Rogers, D.; Hahn, M. Extended-Connectivity Fingerprints. *J. Chem. Inf. Model.* **2010**, *50* (5), 742–754. https://doi.org/10.1021/ci100050t.

(13) Kearnes, S.; McCloskey, K.; Berndl, M.; Pande, V.; Riley, P. Molecular Graph Convolutions: Moving beyond Fingerprints. *J. Comput. Aided. Mol. Des.* **2016**, *30* (8), 595–608. https://doi.org/10.1007/s10822-016-9938-8.

(14) Duvenaud, D.; Maclaurin, D.; Aguilera-iparraguirre, J.; Rafael, G.; Hirzel, T.; Adams, R. P. Convolutional Networks on Graphs for Learning Molecular Fingerprints. *arXiv* **2015**, *arXiv:1509*, 1–9.

(15) Riniker, S.; Landrum, G. A. Open-Source Platform to Benchmark Fingerprints for Ligand-Based Virtual Screening. *J. Cheminform.* **2013**, *5* (26), 1–17.

(16) Cano, G.; Garcia-Rodriguez, J.; Garcia-Garcia, A.; Perez-Sanchez, H.; Benediktsson, J. A.; Thapa, A.; Barr, A. Automatic Selection of Molecular Descriptors Using Random Forest: Application to Drug Discovery. *Expert Syst. Appl.* **2017**, *72*, 151–159. https://doi.org/10.1016/j.eswa.2016.12.008.

(17) Yu, H.; Chen, J.; Xu, X.; Li, Y.; Zhao, H.; Fang, Y.; Li, X.; Zhou, W.; Wang, W.; Wang, Y. A Systematic Prediction of Multiple Drug-Target Interactions from Chemical , Genomic , and Pharmacological Data. *PLoS One* **2012**, *7* (5), 1–14. https://doi.org/10.1371/journal.pone.0037608.

(18) Bleakley, K.; Yamanishi, Y. Supervised Prediction of Drug-Target Interactions Using Bipartite Local Models. *Bioinformatics* **2009**, *25* (18), 2397–2403. https://doi.org/10.1093/bioinformatics/btp433.

(19) Emig, D.; Ivliev, A.; Pustovalova, O.; Lancashire, L.; Bureeva, S.; Nikolsky, Y.; Bessarabova, M. Drug Target Prediction and Repositioning Using an Integrated Network-Based Approach. *PLoS One* **2013**, *8* (4), 1–17. https://doi.org/10.1371/journal.pone.0060618.

(20) Lecun, Y.; Bengio, Y.; Hinton, G. Deep Learning. *Nature* **2015**, *521*, 436–444. https://doi.org/10.1038/nature14539.

(21) Hinton, G.; Deng, L.; Yu, D.; Dahl, G. E.; Mohamed, A.; Jaitly, N.; Senior, A.; Vanhoucke, V.; Nguyen, P.; Sainath, T. N.; et al. Deep Neural Networks for Acoustic Modeling in Speech Recognition. *IEEE Signal Process. Mag.* **2012**, *29* (6), 82–97. https://doi.org/10.1109/MSP.2012.2205597.

(22) Ma, J.; Sheridan, R. P.; Liaw, A.; Dahl, G. E.; Svetnik, V. Deep Neural Nets as a Method for Quantitative Structure − Activity Relationships. *J. Chem. Inf. Model.* **2015**, *55*, 263−274. https://doi.org/10.1021/ci500747n.

(23) Lenselink, E. B.; Ten Dijke, N.; Bongers, B.; Papadatos, G.; Van Vlijmen, H. W. T.; Kowalczyk, W.; Ijzerman, A. P.; Van Westen, G. J. P. Beyond the Hype: Deep Neural Networks Outperform Established Methods Using a ChEMBL Bioactivity Benchmark Set. *J. Cheminform.* **2017**, *9* (45), 1–14. https://doi.org/10.1186/s13321-017-0232-0.

(24) Wallach, I.; Dzamba, M.; Heifets, A. AtomNet : A Deep Convolutional Neural Network for Bioactivity Prediction in Structure-Based Drug Discovery. *arXiv* **2015**, *arXiv:1510*, 1–11.

(25) Ragoza, M.; Hochuli, J.; Idrobo, E.; Sunseri, J.; Koes, D. R. Protein-Ligand Scoring with Convolutional Neural Networks. *J. Chem. Inf. Model.* **2017**, *57* (4), 942–957. https://doi.org/10.1021/acs.jcim.6b00740.

(26) Gonczarek, A.; Tomczak, J. M.; Zaręba, S.; Kaczmar, J.; Dąbrowski, P.; Walczak, M. J. Interaction Prediction in Structure-Based Virtual Screening Using Deep Learning. *Comput. Biol. Med.* **2017**, *100* (September), 253–258. https://doi.org/10.1016/j.compbiomed.2017.09.007.

(27) Altae-Tran, H.; Ramsundar, B.; Pappu, A. S.; Pande, V. Low Data Drug Discovery with One-

Shot Learning. *ACS Cent. Sci.* **2017**, *3* (4), 283–293. https://doi.org/10.1021/acscentsci.6b00367.

(28) Ramsundar, B.; Kearnes, S.; Edu, K.; Riley, P.; Webster, D.; Konerding, D.; Pande, V. Massively Multitask Networks for Drug Discovery. *arXiv* **2015**, 1–27. https://doi.org/https://arxiv.org/abs/1502.02072.

(29) Unterthiner, T.; Mayr, A.; Klambauer, G.; Steijaert, M.; Wegner, J. K.; Ceulemans, H. Deep Learning as an Opportunity in Virtual Screening. *Deep Learn. Represent. Learn. Work. NIPS 2014* **2014**, 1–9.

(30) Sheridan, R. P.; Kearsley, S. K. Why Do We Need so Many Chemical Simialrity Search Methods? *Ddt* **2002**, *7* (17), 903–911.

(31) Cheng, H.-T.; Koc, L.; Harmsen, J.; Shaked, T.; Chandra, T.; Aradhye, H.; Anderson, G.; Corrado, G.; Chai, W.; Ispir, M. Wide & Deep Learning for Recommender Systems. *Proc. 1st Work. Deep Learn. Recomm. Syst.* **2016**, 7–10. https://doi.org/10.1145/2988450.2988454.

(32) Mitchell, A. L.; Attwood, T. K.; Babbitt, P. C.; Blum, M.; Bork, P.; Bridge, A.; Brown, S. D.; Chang, H.; El-gebali, S.; Fraser, M. I.; et al. InterPro in 2019 : Improving Coverage , Classification and Access to Protein Sequence Annotations. *Nucleic Acids Res.* **2019**, *47* (Database Issue), 351–360. https://doi.org/10.1093/nar/gky1100.

(33) Doğan, T.; MacDougall, A.; Saidi, R.; Poggioli, D.; Bateman, A.; O'Donovan, C.; Martin, M. J. UniProt-DAAC: Domain Architecture Alignment and Classification, a New Method for Automatic Functional Annotation in UniProtKB. *Bioinformatics* **2016**, *32* (15), 2264–2271. https://doi.org/10.1093/bioinformatics/btw114.

(34) Bateman, A.; Martin, M.-J.; Orchard, S.; Magrane, M.; Alpi, E.; Bely, B.; Bingley, M.; Britto, R.; Bursteinas, B.; Busiello, G.; et al. UniProt: A Worldwide Hub of Protein Knowledge. *Nucleic Acids Res.* **2018**, *47* (D1), D506–D515. https://doi.org/10.1093/nar/gky1049.

(35) Hamosh, A.; Scott, A. F.; Amberger, J. S.; Bocchini, C. A.; Mckusick, V. A. Online Mendelian Inheritance in Man (OMIM), a Knowledgebase of Human Genes and Genetic

Disorders. *Nucleic Acids Res.* **2005**, *33* (Database), 514–517. https://doi.org/10.1093/nar/gki033.

(36) Rath, A.; Olry, A.; Dhombres, F.; Mili, M.; Urbero, B.; Ayme, S. Representation of Rare Diseases in Health Information Systems : The Orphanet Approach to Serve a Wide Range. *Hum. Mutat.* **2012**, *33* (5), 803–808. https://doi.org/10.1002/humu.22078.

(37) Mikołajczyk, A.; Grochowski, M. Data Augmentation for Improving Deep Learning in Image Classification Problem. *2018 Int. Interdiscip. PhD Work.* **2018**, 117–122. https://doi.org/10.1109/IIPHDW.2018.8388338.

(38) Thomas, N.; Smidt, T.; Kearnes, S.; Yang, L.; Li, L.; Kohlhoff, K.; Riley, P. Tensor Field Networks : Rotation- and Translation-Equivariant Neural Networks for 3D Point Clouds. *arXiv* **2018**, *1802.08219*, 1–19.

Supplementary Figures

(a)

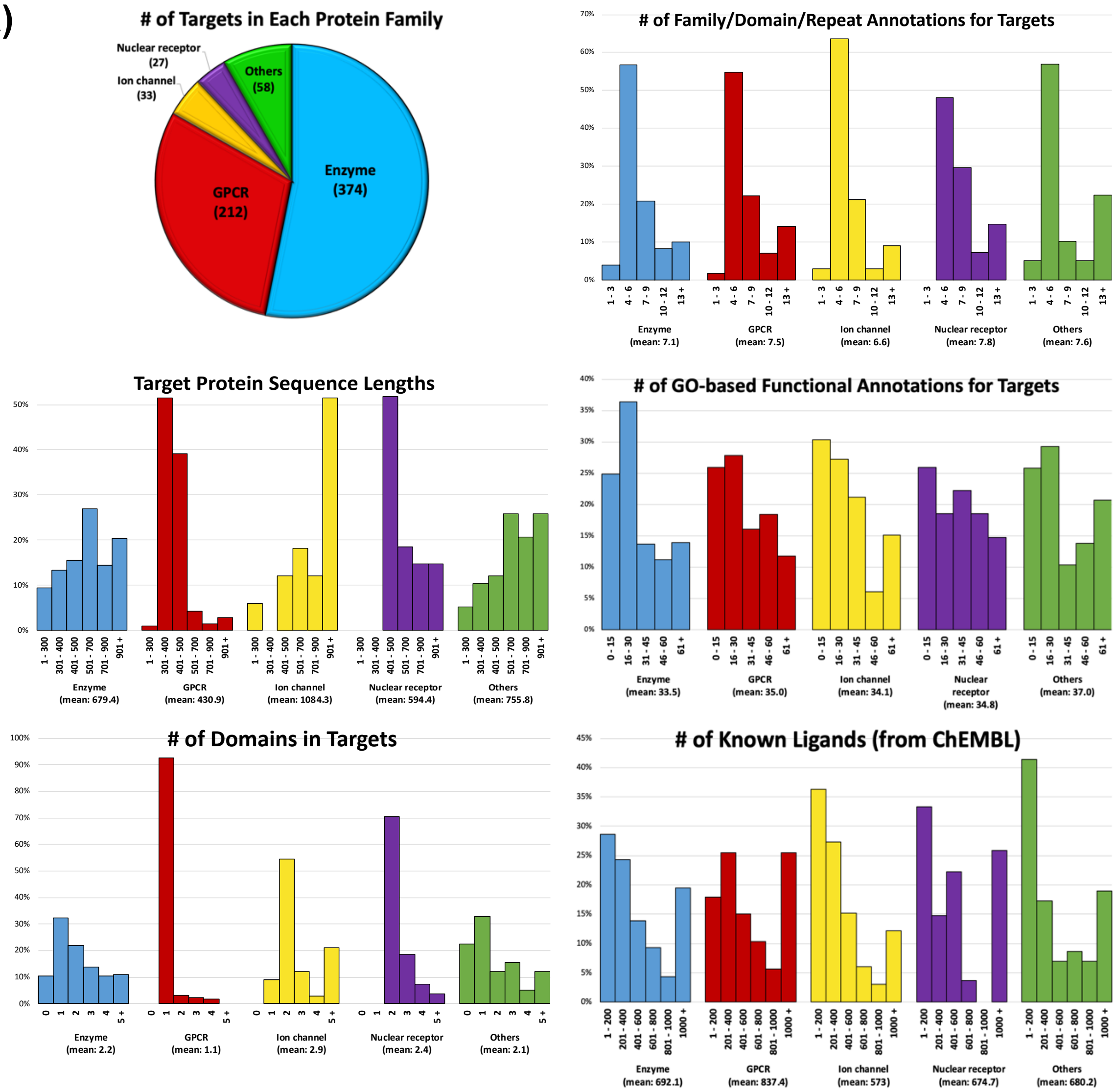

(b)

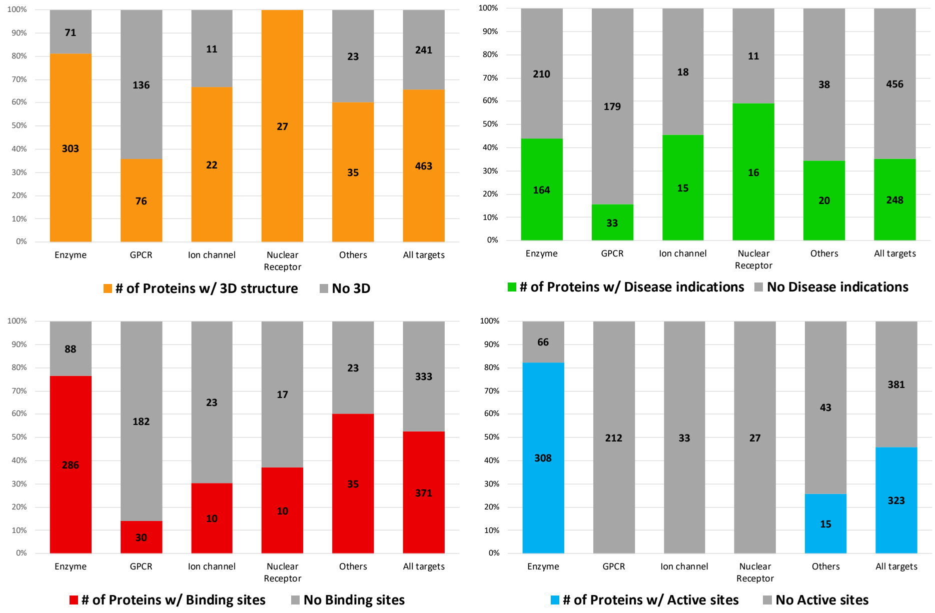

Figure S.1 Protein family-based annotation analysis of DEEPScreen targets. **(a)** Target protein family size pie chart and the histograms for the distributions of the protein lengths, number of structural domains, number of family/domain/repeat annotations, number of GO-based functional annotations and the number of known small molecule ligands. **(b)** Bar graphs depicting the number of proteins that possess 3-D structure, binding-active site and disease indication annotations.

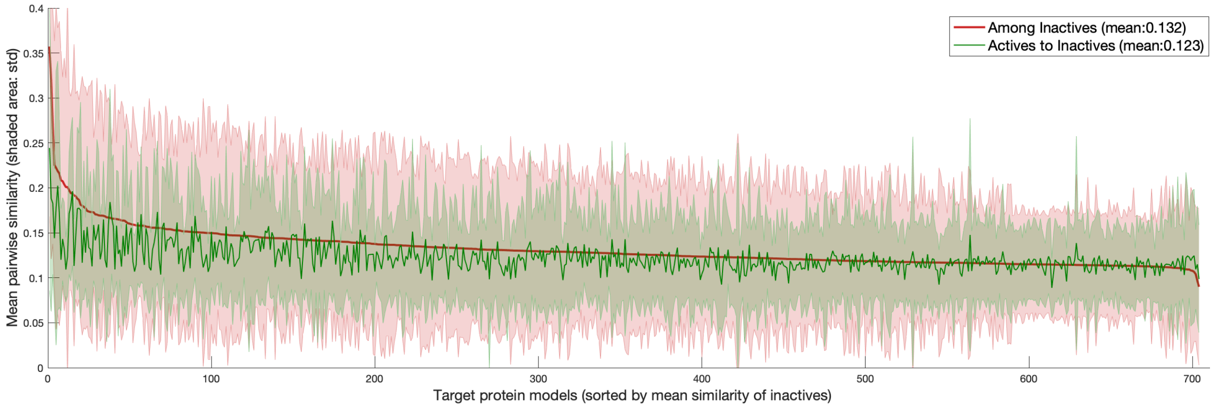

Figure S.2 Target-based (x-axis) average pairwise compound similarity (y-axis) curves for intra-group (among inactives) and inter-group (actives to inactives) similarities with error bands for 704 DEEPScreen targets.

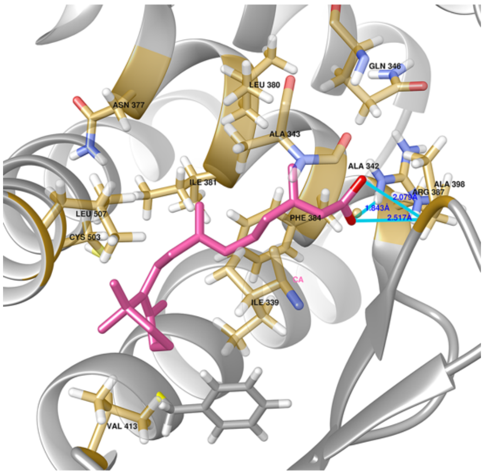

Figure S.3 3-D structural representation of the molecular docking (best pose) of tretinoin – retinoic acid receptor RXRBeta pair. Molecular docking was employed since tretinoin – RXRBeta complex have not been structurally characterized, although the interaction is part of an approved medication. However, the X-ray crystal structure of Tretinoin - RAR RXR-gamma complex (another DTI from the same approved medication, where the target has an equivalent 3-D structure to RXRbeta) is found in PDB (id: 2LBD). The known binding site of RXRbeta are shown in gold color, and was extracted from PDBid: 1UHL. Tretinoin is displayed in magenta and the hydrogen bonds as light blue lines. The resulting binding free energy ΔG = -11.4 kj/mol (Kd ≈ 4.1 nM) at the best pose, together with the median experimental screening assay activity Ki = 54 nM obtained from the ChMEBL database, indicates high potency for tretinoin against RXRbeta.

Supplementary Tables

Table S.1: Hyper-parameter types and the tested values during the training of DEEPScreen.

Hyper-parameter Name	Test values
Input Normalization	Yes
	No
Learning rate	0.0005
	0.0001
	0.005
	0.001
	0.01
Filter size	3
	5
Stride	1
Padding	"same"
Number of convolutional layers	*
Number of filters in each convolutional layer	**
Number of neurons in each fully-connected layer	***
Optimizer	Adam (default)
	Momentum (default)
	RMSprop (default)
Mini-batch size	32
	64
Drop-out rate	0.5
	0.6
	0.8
Batch Normalization	Yes

* Values between 3 and 8 were tested for the in-house DCNN architecture. For AlexNET and Inception, the default architectures were directly used without any change.

** Numerous "# of filter" value combinations were tested between 16 and 256.

*** For the two fully-connected layers (just before the output layer) in AlexNET, the number of neurons that were tested: (128,16), (256,128), (512,32), (1024,32) and (2048,2048). For the in-house DCNN, there were one fully connected-layer (before the output layer) and # of neurons tested were: 16, 32, 128, 256 and 512.

Table S.2: The performance results and the computational requirements (in training) of 3 target protein models in the input image size analysis.

	Input Image Size		
**Test performance results of the best model (average of 3 target protein models):**	**100x100**	**200x200**	**400x400**
MCC	0.59	0.69	0.65
F1-score	0.79	0.84	0.83
Accuracy	0.79	0.84	0.83
Precision	0.83	0.87	0.84
Recall	0.76	0.83	0.83
**Computational requirements for in-house DCNN and Inception model training (average of 3 target protein models):**			
CNNModel run time (min)	8	46	192
Inception run time (min)	75	470	-
CNNModel memory (Gb)	0.7	2.6	7.9
Inception memory (Gb)	3.3	7.3	-

Table S.3: Cytotoxicity of Cladribine on HCC cell lines.

Cancer Cells	24 hrs		48 hrs		72hrs	
	**IC_{50}**	**R^{2*}**	**IC_{50}**	**R^{2*}**	**IC_{50}**	**R^{2*}**
**Huh7**	>40 µM	NA	>40 µM	0,92	3 µM	0,97
**HepG2**	>40 µM	NA	7 µM	0,92	0.1 µM	0,84
**Mahlavu**	21 µM	0,9	3 µM	0,92	0.4 µM	0,93

* Experiments are done in triplicates.

Table S.4: DEEPScreen target predictions for Cladribine.

Target ChEMBL ID	Target UniProt Acc.	Target Entry Name	Target Gene Name	Target Organism	Drug ChEMBL ID	Drug Name	CAS Number	Model Score
CHEMBL6115	P10619	PPGB	CTSA	HUMAN	CHEMBL1619	Cladribine	4291-63-8	0.9
CHEMBL4979	P13866	SC5A1	SLC5A1	HUMAN	CHEMBL1619	Cladribine	4291-63-8	0.75
CHEMBL4803	P29474	NOS3	NOS3	HUMAN	CHEMBL1619	Cladribine	4291-63-8	0.56
CHEMBL4701	P31390	HRH1	HRH1	RAT	CHEMBL1619	Cladribine	4291-63-8	0.78
CHEMBL4588	P22894	MMP8	MMP8	HUMAN	CHEMBL1619	Cladribine	4291-63-8	0.64
CHEMBL4523	Q9P1W9	PIM2	PIM2	HUMAN	CHEMBL1619	Cladribine	4291-63-8	0.8
CHEMBL4481	P35228	NOS2	NOS2	HUMAN	CHEMBL1619	Cladribine	4291-63-8	0.59
CHEMBL4079	P25098	ARBK1	GRK2	HUMAN	CHEMBL1619	Cladribine	4291-63-8	0.85
CHEMBL3884	P31639	SC5A2	SLC5A2	HUMAN	CHEMBL1619	Cladribine	4291-63-8	0.84
CHEMBL3863	Q15139	KPCD1	PRKD1	HUMAN	CHEMBL1619	Cladribine	4291-63-8	0.75
CHEMBL3571	P20272	CNR1	CNR1	RAT	CHEMBL1619	Cladribine	4291-63-8	0.85
CHEMBL3535	O76083	PDE9A	PDE9A	HUMAN	CHEMBL1619	Cladribine	4291-63-8	0.84
CHEMBL3529	Q14164	IKKE	IKBKE	HUMAN	CHEMBL1619	Cladribine	4291-63-8	0.74
CHEMBL3478	Q28156	PDE5A	PDE5A	BOVIN	CHEMBL1619	Cladribine	4291-63-8	0.71
CHEMBL3474	P14555	PA2GA	PLA2G2A	HUMAN	CHEMBL1619	Cladribine	4291-63-8	0.72
CHEMBL3426	P47898	5HT5A	HTR5A	HUMAN	CHEMBL1619	Cladribine	4291-63-8	0.65
CHEMBL3374	P25104	AGTR1	AGTR1	BOVIN	CHEMBL1619	Cladribine	4291-63-8	0.84
CHEMBL330	P35439	NMDZ1	GRIN1	RAT	CHEMBL1619	Cladribine	4291-63-8	0.74
CHEMBL3286	P00749	UROK	PLAU	HUMAN	CHEMBL1619	Cladribine	4291-63-8	0.59
CHEMBL3268	Q9UBF8	PI4KB	PI4KB	HUMAN	CHEMBL1619	Cladribine	4291-63-8	0.43
CHEMBL3142	P78527	PRKDC	PRKDC	HUMAN	CHEMBL1619	Cladribine	4291-63-8	0.62
CHEMBL3138	P19020	DRD3	DRD3	RAT	CHEMBL1619	Cladribine	4291-63-8	0.85
CHEMBL3045	P05771	KPCB	PRKCB	HUMAN	CHEMBL1619	Cladribine	4291-63-8	0.83
CHEMBL2996	Q05655	KPCD	PRKCD	HUMAN	CHEMBL1619	Cladribine	4291-63-8	0.83
CHEMBL2971	O60674	JAK2	JAK2	HUMAN	CHEMBL1619	Cladribine	4291-63-8	0.74
CHEMBL2835	P23458	JAK1	JAK1	HUMAN	CHEMBL1619	Cladribine	4291-63-8	0.81
CHEMBL280	P45452	MMP13	MMP13	HUMAN	CHEMBL1619	Cladribine	4291-63-8	0.65
CHEMBL275	Q07343	PDE4B	PDE4B	HUMAN	CHEMBL1619	Cladribine	4291-63-8	0.56
CHEMBL2664	P23526	SAHH	AHCY	HUMAN	CHEMBL1619	Cladribine	4291-63-8	1
CHEMBL263	P29089	AGTRB	AGTR1B	RAT	CHEMBL1619	Cladribine	4291-63-8	0.93
CHEMBL262	P49841	GSK3B	GSK3B	HUMAN	CHEMBL1619	Cladribine	4291-63-8	0.55
CHEMBL2335	P42785	PCP	PRCP	HUMAN	CHEMBL1619	Cladribine	4291-63-8	0.88
CHEMBL2334	P42574	CASP3	CASP3	HUMAN	CHEMBL1619	Cladribine	4291-63-8	0.58
CHEMBL2276	P45983	MK08	MAPK8	HUMAN	CHEMBL1619	Cladribine	4291-63-8	0.62
CHEMBL2216739	Q92523	CPT1B	CPT1B	HUMAN	CHEMBL1619	Cladribine	4291-63-8	0.68
CHEMBL2148	P52333	JAK3	JAK3	HUMAN	CHEMBL1619	Cladribine	4291-63-8	0.72
CHEMBL2107	P61073	CXCR4	CXCR4	HUMAN	CHEMBL1619	Cladribine	4291-63-8	0.95
CHEMBL209	P07477	TRY1	PRSS1	HUMAN	CHEMBL1619	Cladribine	4291-63-8	0.6
CHEMBL2014	P41146	OPRX	OPRL1	HUMAN	CHEMBL1619	Cladribine	4291-63-8	0.85
CHEMBL1991	O14920	IKKB	IKBKB	HUMAN	CHEMBL1619	Cladribine	4291-63-8	0.66
CHEMBL1981	P06213	INSR	INSR	HUMAN	CHEMBL1619	Cladribine	4291-63-8	0.69
CHEMBL1899	P46098	5HT3A	HTR3A	HUMAN	CHEMBL1619	Cladribine	4291-63-8	0.64
CHEMBL1875	Q13639	5HT4R	HTR4	HUMAN	CHEMBL1619	Cladribine	4291-63-8	0.79
CHEMBL1865	Q9UBN7	HDAC6	HDAC6	HUMAN	CHEMBL1619	Cladribine	4291-63-8	0.5

Table S.5: Pairwise all-against-all Spearman rank correlation matrix comparing model performance (MCC) ranks of DEEPScreen, LR-ECFP, RF-ECFP, SVM-ECFP, LR-Image, RF-Image and SVM-Image.

	**DEEPScreen**	**LR-ECFP**	**RF-ECFP**	**SVM-ECFP**	**LR-Image**	**RF-Image**	**SVM-Image**
**DEEPScreen**		0.241	0.252	0.512	0.685	0.645	0.330
**LR-ECFP**	0.241		0.968	0.604	0.213	0.202	0.136
**RF-ECFP**	0.252	0.968		0.636	0.231	0.222	0.173
**SVM-ECFP**	0.512	0.604	0.636		0.521	0.521	0.321
**LR-Image**	0.685	0.213	0.231	0.521		0.802	0.245
**RF-Image**	0.645	0.202	0.222	0.521	0.802		0.333
**SVM-Image**	0.330	0.136	0.173	0.321	0.245	0.333	
